# Prevalence, risk factors, and treatment methods of thirst in critically ill patients: A systematic review and meta-analysis

**DOI:** 10.1371/journal.pone.0315500

**Published:** 2025-03-18

**Authors:** Takuto Fukunaga, Akira Ouchi, Gen Aikawa, Saiko Okamoto, Shogo Uno, Hideaki Sakuramoto

**Affiliations:** 1 Department of Emergency and Critical Care Medicine, Toho University Omori Medical Center, Ota-ku, Tokyo, Japan; 2 Department of Adult Health Nursing, College of Nursing, Ibaraki Christian University, Hitachi, Ibaraki, Japan; 3 College of Nursing, Kanto Gakuin University, Yokohama, Kanagawa, Japan; 4 Department of Emergency and Critical Care Medicine, Hitachi General Hospital, Hitachi, Ibaraki, Japan; 5 Department of Critical Care and Disaster Nursing, Japanese Red Cross Kyushu International College of Nursing, Munakata, Fukuoka, Japan; Satyawati College, University of Delhi, INDIA

## Abstract

Critically ill patients admitted to the intensive care unit (ICU) experience various symptoms and discomfort. Although thirst is a typical distressing symptom and should be assessed daily, it is crucial to understand its prevalence and risk factors in the ICU setting. Nevertheless, currently, systematic reviews of prevalence and risk factors are lacking. This study evaluated the prevalence and risk factors of thirst in critically ill patients. We conducted a comprehensive search of the MEDLINE, Cochrane Library, and CINAHL databases. The study design included cohort, cross-sectional, and intervention studies, including randomized and non-randomized controlled trials with control groups. The point estimates from each study were combined using a random-effects meta-analysis model. We aggregated the prevalence of thirst in ICU patients and calculated the point estimates and 95% confidence intervals. The risk of bias was assessed using the Cochrane Risk of Bias 2 tool and Newcastle-Ottawa Scale. Fifteen studies were eligible for inclusion, of which seven reported the prevalence of thirst. A total of 2,204 patients were combined, with a prevalence estimate of 0.70. The risk factors for thirst were categorized as patient and treatment factors: four patient factors (e.g., serum sodium concentration and severity of illness) and six treatment factors (e.g., nil per os and use of diuretics) were identified. However, the results showed high heterogeneity in the prevalence of thirst among critically ill patients. It was established that 70% of critically ill patients experienced thirst. Additional investigations are required to obtain a more comprehensive overview of thirst among these patients.

Systematic review registration number

The protocol was registered in PROSPERO (ID: CRD42023428619) on June 6, 2023. (URL: https://www.crd.york.ac.uk)

## Introduction

Critically ill patients in the intensive care unit (ICU) experience various symptoms and discomfort [1,2], and recently, thirst has been highlighted as a stressful symptom. In their study, Chanques et al. [3] reported that thirst is one of five ICU patient symptoms that should be evaluated daily because it is the most prevalent and intense symptom reported by ICU patients. This statement is supported by various studies that highlight that thirst is related to significant distress and stress, increased oxygen consumption and metabolic load on organs [4,5], and induced delirium [6], which all affect recovery.

The prevalence of thirst in critically ill patients varies between studies. Some studies [3,4] reported a prevalence of thirst in severely ill patients ranging from 40–70%. However, there are no meta-analyses addressing thirst in critically ill patients and therefore the prevalence is unknown.

In addition, risk factors and treatment methods for thirst in critically ill patients remain unclear. Thirst is associated with xerostomia, endotracheal tubes, tracheostomies, oxygen therapy, solid and liquid fasting, electrolyte alterations, and hypovolemia. In a study conducted in three ICUs in a tertiary medical center [7], oral swab wipes, sterile ice water spray, and lip balm significantly reduced thirst intensity and distress. In a survey of 61 ICU patients [8], vitamin C sprays, peppermint water mouthwash, and a lip moisturizer significantly reduced thirst intensity. Both studies recommended combining these measures as an oral care bundle. However, few studies have examined interventions for thirst, and risk factors and effective treatments are unknown.

Therefore, we conducted a systematic review and meta-analysis of thirst in critically ill patients to examine the prevalence and risk factors thereof. The secondary aim was to identify assessment and treatment methods. Our findings will contribute to a better clinical understanding of thirst in critically ill patients.

## Materials and methods

### Protocol and registration

This systematic review and meta-analysis examined the prevalence of thirst, assessment methods, risk factors, and symptom relief methods in critically ill patients. The study methodology followed the Preferred Reporting Items for Systematic Reviews and Meta-Analyses Statement [9], and a systematic review was conducted. The protocol was registered in PROSPERO (ID: CRD42023428619) on June 6, 2023 (S1 File). The systematic review adhered to the PRISMA 2020 checklist (S2 File).

### Literature search strategy

We searched for articles in three electronic databases: MEDLINE via PubMed, the Cochrane Library (CENTRAL), and CINAHL. We also conducted a manual search using Google Scholar. All English publications published until June 6, 2024 were searched without any restrictions on countries. The reference lists of all selected articles were independently screened to identify additional studies that were excluded from the initial search. The search formulae for PubMed, CENTRAL, and CINAHL are presented in Supplementary S3 File.

### Study screening and selection

Two of the five reviewers (TF, GA, SO, SU, and HS) independently screened the titles and abstracts of all studies to identify potentially relevant studies. Subsequently, the full texts were independently reviewed according to a standardized protocol. Any disagreements between the two reviewers were resolved through discussion, and if necessary, a third reviewer (AO) was brought in for arbitration.

### Eligibility criteria

To conduct a comprehensive and exploratory review of thirst in critically ill patients, we used the “population–concept–context” framework [10] recommended by the Joanna Briggs Institute as a guide for developing clear and meaningful objectives and eligibility criteria; The study population included critically ill adult patients expected to stay in the ICU for more than 24 hours, the concept was any treatment for thirst or dry mouth, and the context was critically ill patients in ICUs. The study design included cohort, cross-sectional, and prospective studies, including randomized controlled trials (RCTs) and non-RCTs (NRCTs) with control groups. Only studies reporting the prevalence of thirst, risk factors, and treatment methods for thirst in critically ill patients, respectively; that did not include preclinical results; were in English; and that included human participants were included.

### Data extraction and quality assessment

The prevalence of thirst was recorded as the primary outcome of each study. Secondary evaluations were collected from the literature and included assessment methods and risk factors for thirst, and symptom relief methods. Data extraction was performed by TF. Data were extracted using Microsoft Excel. Each article was summarized under the following data headings in an Excel spreadsheet: First author; Year of publication; Title of Article; Study characteristics; Sample size; Control group; Prevalence; Demographic factors (including average age, severity of illness, etc.); Symptom relief methods; Risk factors; Assessment methods; and Definition of thirst.

The risk of bias in the target literature was assessed using the Cochrane Risk of Bias 2 tool (RoB2) for RCTs [11], and the study design’s quality and degree of potential bias were assessed according to the RoB2 domains. Two reviewers (TF and AO) independently evaluated the risk of bias using the RoB2. Disagreements between the two reviewers were discussed and if unresolved, a third reviewer (HS) was brought in for arbitration. The Newcastle-Ottawa Scale was used for observational studies, cross-sectional studies, and NRCTs. For RCTs, we assessed each of the following six domains: (1) selection bias in the process of randomization assignment, (2) selection bias in the process of concealment of assignment, (3) implementation bias in the process of blinding to participants and interventionists, (4) measurement bias in the process of blinding to outcome assessors and analysts, (5) missing bias due to participant attrition, and (6) reporting bias in the reporting of outcomes were assessed. For observational studies, cross-sectional studies, and NRCTs, we assessed each of the following three domains: (1) representativeness, exposure assessment, etc. (four items), (2) confounding assessment (two items), and (3) outcomes (three items). For observational studies, cross-sectional studies, and NRCTs, the risk of bias was assessed according to each of the following three domains: (1) representativeness, exposure assessment, etc. (four items), (2) confounding assessment (two items), and (3) outcomes (three items). The risk of bias for the Newcastle-Ottawa Scale was assessed by summing the scores of the nine criteria to evaluate the overall quality of each study; if the two raters had different opinions, a decision was made through an inter-rater discussion.

### Statistical analysis

Analyses were conducted via Stata/BE (version 18.0; Stata Corp., College Station, TX, USA). The point estimates from each study were combined using a random-effects meta-analysis model to obtain an overall estimate using the Der Simonian–Laird method. The Freeman–Tukey double-arcsine transformation was chosen as an effect size measure. The estimate was considered significant when the Z test p-value was < .05. We assessed heterogeneity with Cochrane’s Q test and tau-squared (T^2^) and measured the inconsistency (the percentage of total variation across studies due to heterogeneity) of effects across interventions using the I^2^ statistic. Publication bias was assessed using a funnel plot and Egger’s test, with significance set at P < .05. In addition, the prevalence of each definition of thirst sensation was extracted by subgroup analysis. A meta-regression analysis to determine the contribution of specific cofactors (mean age, sex ratio, mechanical ventilator use, and length of ICU stay) to heterogeneity was conducted.

## Results

### Literature search results

**Fig 1:**
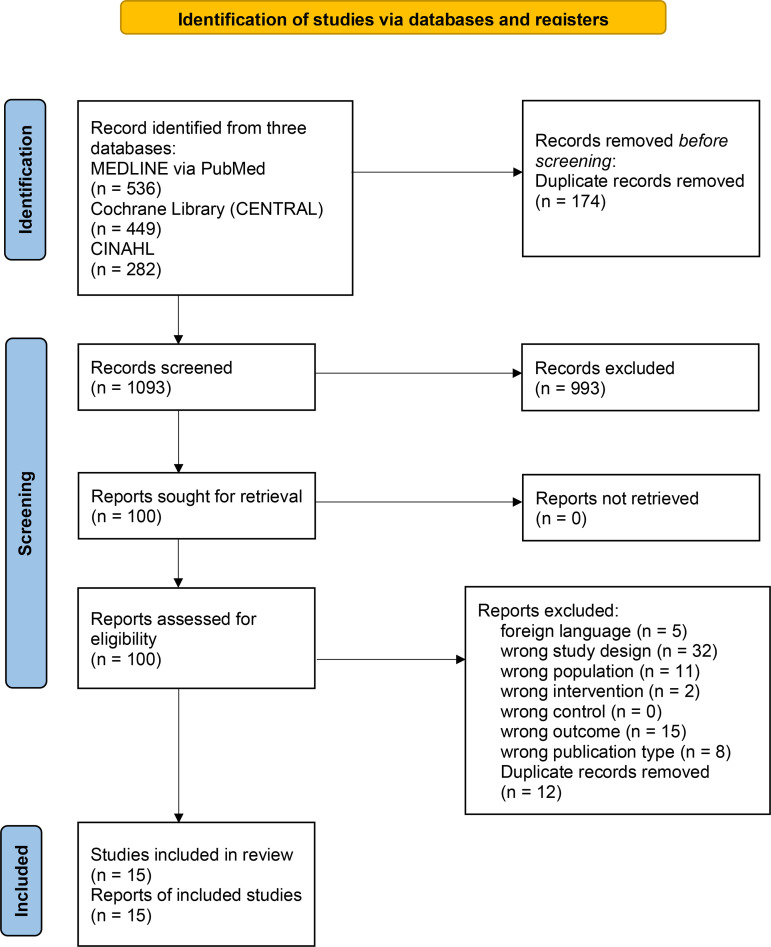
Preferred reporting items for systematic reviews and meta-analyses statement flowchart.

The literature search results are summarized in Fig 1. A total of 1,267 references were retrieved from the three English databases. A total of 1,093 references were targeted for primary screening after excluding duplicates. Subsequently, 100 studies were selected and evaluated for full text as secondary screening. Fifteen eligible studies met the inclusion criteria.

### Selected literature characteristics

After the screening, 15 studies were selected for eligibility. Regarding the study design, nine studies were observational [4,12–19], two studies were cross-sectional [6,20], and four studies were RCTs [7,8,21,22]. Six studies were from the United States [4,7,12,13,16,20], two each from France [14,22], Japan [6,15], and China [8,18], and one each from Italy [17], South Korea [21], and Norway [19]. Two references were published in the 2000s [12,13], seven in the 2010s [4,6,7,14,16,20,21], and six in the 2020s [8,15,17–19,22].

Regarding patient characteristics of the selected references, five studies included only patients on ventilators [13,16,17,19,21] and four studies had a mean patient age of 65 years or older [6,12,14,15]. Furthermore, eight references were mixed ICUs [6,7,13,15,18–20,22], two were internal medicine ICUs [12,21], three were general ICUs [8,14,17], one was a surgical ICU [16], and another was an ICU with no type description [4]. Some internal medicine ICUs were included in the literature, thus limiting their scope to patients with cancer (Table 1).

**Table 1:** Study characteristics.

Number	Author_year	Country	Study design	Purpose	Inclusion criteria	Key findings
1	Nelson JE_2001 [12]	USA	Prospective observational study	To clarify the experience of symptoms of ICU patients at high risk of in-hospital mortality	Cancer patients admitted to MICU	Using the Edmonton Symptom Assessment Scale, ICU patients at high risk of in-hospital mortality experience pain, discomfort, anxiety, sleep disturbances, and hunger and thirst
2	Li DTY_2007 [13]	USA	Prospective, descriptive study	To determine cardiovascular and pupillary reactivity and behavioral responses during distressing and non-distressing procedures in sedated ICU patients	Patients aged between 21 and 80 years Mechanical ventilated for more than 12 hours Understand English Ramsay score of 2	Specific physiological responses and changes in pupil size are potentially useful nociceptive indicators in the ICU setting
3	Puntillo KA_2010 [4]	USA	Prospective, observational study	To assess the symptom experience of ICU patients at high risk of death and evaluate the relationship between delirium and patient symptoms	Patients 18 years or older admitted to the ICU for at least 3 days APACHE II score of ≧ 20 in the first 24 hours One or more of the following diagnoses: acute heart failure, respiratory failure, chronic liver failure with cirrhosis, multiorgan failure, sepsis, or systemic failure associated with a diagnosis of malignancy	Patients at high risk of death experienced pain, tiredness, shortness of breath, restlessness, anxiousness, sadness, hunger, scared, thirst and confusion with a prevalence of thirst of 70.8 Patients with delirium were significantly more likely to report confusion (43% vs. 22%, p = .004) and sadness (46% vs. 31%, p = .04) than those without delirium
4	Siami S_2013 [14]	France	Prospective interventional study	To examine how vasopressin secretion during an osmotic challenge (500 mL of hypertonic saline administered for 120 minutes) is a variable response in patients recovering from septic shock	Adult patients hospitalized with septic shock who survived five days after the discontinuation of vasopressor therapy	60% of patients recovering from septic shock had no response to vasopressin secretion during osmotic challenge
5	Doi S_2021 [15]	Japan	Cohort study	To investigate the impact of oral care on the assessment of thirst and dry mouth in ICU patients	Patients ≧ 18 years of age	The NRS for thirst decreased from immediately to 1 hour after oral care
6	Duffy EI_2018 [16]	USA	Pilot prospective study	Validate devices that aid communication, such as communication boards and eye tracking devices	Mechanical ventilated patients Speaks English No cognitive impairment No traumatic brain injury with motor impairment	The median accuracy of the communication board was (100% [IQR 100%-100%]) and the eye tracking device had a median accuracy of (100% [IQR 68.8%-100%])
7	Jang CS_2016 [21]	South Korea	Randomized clinical trial	To evaluate the combined effect of oral healthcare on oral health status, dry mouth, and salivary pH in critically ill and intubated patients	Intubated patients over 20 years of age	Beck Oral Health Assessment Scale: (8.1 vs. 11.1, P < .001) Dry mouth (sublingual gland): (2.0 vs. 1.0, P < .001) Dry mouth (sublingual gland):(1.0 vs. 0.0, P < .001) Saliva pH: (6.5 vs. 5.7, P < .001)
8	Puntillo KA_2014 [7]	USA	Single-blinded, randomized clinical trial	To examine the effects of intervention bundles on thirst intensity, thirst distress, and dry mouth	Patients aged over 18 years ICU stay for more than 24 hours Speaks English Can give name, date and location RASS of -1 to + 1 Either thirst intensity or thirst distress score at screening is NRS 3 or higher	Comparing the mean NRS estimates for the control and intervention groups, the intensity of thirst was 3.6 vs. 4.7 and the distress of thirst was 3.2 vs. 3.7
9	Stotts NA_2015 [20]	USA	Descriptive cross-sectional study	To identify predictors of presence, intensity, and distress of dry mouth in ICU patients	Patients over 18 years of age ICU stay for more than 24 hours Speaks English Can give name, date, and location RASS of -1 to + 1 NRS of thirst is more than 3	Predictors for the presence of thirst were APACHE II (P = .030), NPO (P = .006), high-dose opioid use (P = .001), high-dose furosemide use (P = .038), selective serotonin reuptake inhibitor use (P = .048) and ionized calcium (P = .008) Predictors for thirst intensity were oral intake (P = .001), gastrointestinal disease (P = .020), and NPO (P = .001) Predictors for thirst distress were mechanical ventilator (P = .014), fluid balance (P = .043), antihypertensive medication (P = .001), gastrointestinal disease (P = .003), and NPO (P = .002)
10	Sato K_2019 [6]	Japan	Single-center retrospective cross-sectional study	To determine if persistent severe thirst is strongly associated with the onset of delirium	Patients ≧ 18 years of age RASS of -1 to + 1	The prevalence of strong thirst with NRS 8 or higher was 40.6%, and strong thirst lasting more than 24 hours was associated with delirium (odds ratio, 5.74; 95% confidence interval, 2.53-12.99)
11	Negro A_2022 [17]	Italy	Prospective observational study	To investigate the incidence of thirst sensation in ICU patients and assess the association between dry mouth sensation and endotracheal tube, tracheostomy, spontaneous breathing, and oxygen therapy with or without humidification	Patients 18 years of age or older with tracheal intubation or tracheostomy and spontaneous respiration GCS greater than or equal to 9	The incidence of thirst in ICU patients is as high as 76.1% and is associated with high-dose diuretics, NPO, and dry mouth
12	Merliot-Gailhoustet L_2022 [22]	France	Cross-over randomized controlled trial	To examine the best ways to improve ICU patient discomfort among various electronic relaxation devices	Patients ≧ 18 years of age CAM-ICU negative with RASS greater than or equal to 0 SOFA score of 3 or higher	Virtual reality devices were associated with a significant reduction in overall discomfort (median NRS = 4[2–6] vs. 2[0-5]; p = .01, mixed-effect model), indicating that virtual reality devices, compared to music therapy, were most effective in improving overall discomfort and reducing physiological stress responses in ICU patients. and physiological stress reactions in ICU patients.
13	Zhang W_2022 [8]	China	Prospective, randomized, placebo-controlled	To demonstrate the effectiveness of an intervention bundle to reduce thirst and dry mouth	18 years of age or older ICU stay for more than 24 hours Fasting patients Clear conscious and able to cooperate NRS of thirst measured by screening is greater than or equal to 3	Mean decrease in thirst intensity and objective oral mucosa scale after intervention decreased more for patients in the intervention group compared to controls (-1.27 vs. -0.19, -0.36 vs. -0.1)
14	Lin R_2023 [18]	China	Prospective descriptive design	To analyze the incidence of and factors contributing to dry mouth in ICU patients with and without dry mouth by analyzing differences in physiological, psychological, disease- and environment-related parameters	Patients 18 years of age or older ICU stay for more than 24 hours RASS of -1 to + 1 Able to communicate verbally and understand the questionnaire Consent to participate in the study	The incidence of thirst in ICU patients is as high as 76.1% and is associated with high-dose diuretics, NPO, and dry mouth
15	Saltnes-Lillegård C_2024 [19]	Norway	Prospective cohort study	To describe the prevalence, intensity and distress of five symptoms in ICU patients and to investigate possible predictive factors associated with symptom intensity	Patients 18 years of age or older ICU stay for more than 24 hours Need for mechanical ventilation Need for continuous vasoactive therapy or ICU stay greater than 24 hours	On the first ICU day, thirst as the most prevalent symptom (66%), with the highest mean intensity score On the seven ICU days, thirst as the most prevalent symptom (64%), with the highest mean intensity score During seven days, analgesic administration and sepsis diagnosis were associated with increased thirst intensity

*Note:* ICU, intensive care unit; MICU, medical intensive care unit; RASS, Richmond agitation-sedation scale; APACHE, acute physiologic assessment and chronic health evaluation; SOFA, sequential organ failure assessment; NRS, numerical rating scale; NPO, nil per os.

### Risk of bias assessment

Risk of bias was assessed using the RoB2 and the Newcastle-Ottawa Scale and are summarized in Table 2. Finally, two studies were judged to be high risk, ten to be with some concerns, and three to be low risk.

**Table 2:** Quality rating of studies reporting.

Author_year	Bias tool used	Average Newcastle-Ottawa Scale score (if applicable)	Overall risk of bias
Nelson JE_2001	Newcastle-Ottawa Scale	4	Some concerns
Li DTY_2007	Newcastle-Ottawa Scale	5	Some concerns
Puntillo KA_2010	Newcastle-Ottawa Scale	6	Some concerns
Siami S_2013	Newcastle-Ottawa Scale	7	Low risk
Doi S_2021	Newcastle-Ottawa Scale	7	Low risk
Duffy EI_2018	Newcastle-Ottawa Scale	5	Some concerns
Jang CS_2016	Cochrane Risk of Bias 2		Some concerns
Puntillo KA_2014	Cochrane Risk of Bias 2		Some concerns
Stotts NA_2015	Newcastle-Ottawa Scale	5	Some concerns
Sato K_2019	Newcastle-Ottawa Scale	5	Some concerns
Negro A_2022	Newcastle-Ottawa Scale	3	High risk
Merliot-Gailhoustet L_2022	Cochrane Risk of Bias 2 tool for cross-over trials		High risk
Zhang W_2022	Cochrane Risk of Bias 2		Low risk
Lin R_2023	Newcastle-Ottawa Scale	5	Some concerns
Saltnes-Lillegård C_2024	Newcastle-Ottawa Scale	6	Some concerns

Two reviewers independently assessed the risk of bias for each included study. When the Cochrane Collaboration Risk of Bias Tool was used, the overall risk of bias was determined by following the tool’s standardized instructions: studies assessed to have “low” risk of bias in all tool domains were considered to have “low” overall risk, studies assessed to have “some” risk of bias in at least one tool domain were considered to have “some” overall risk, and studies assessed to have “high” risk of bias in at least one domain *or* assessed to have “some” risk of bias in two or more domains in a way that “substantially lowers confidence in the result” were considered to have “high” overall risk. When the Newcastle-Ottawa Scale was used, the overall risk of bias was determined by the average score of the two reviewers: greater than or equal to seven was considered as having “low” overall risk, greater than or equal to four but less than seven was considered as having “some” overall risk, and less than four was considered having “high” overall risk.

### Primary outcomes

**Fig 2:**
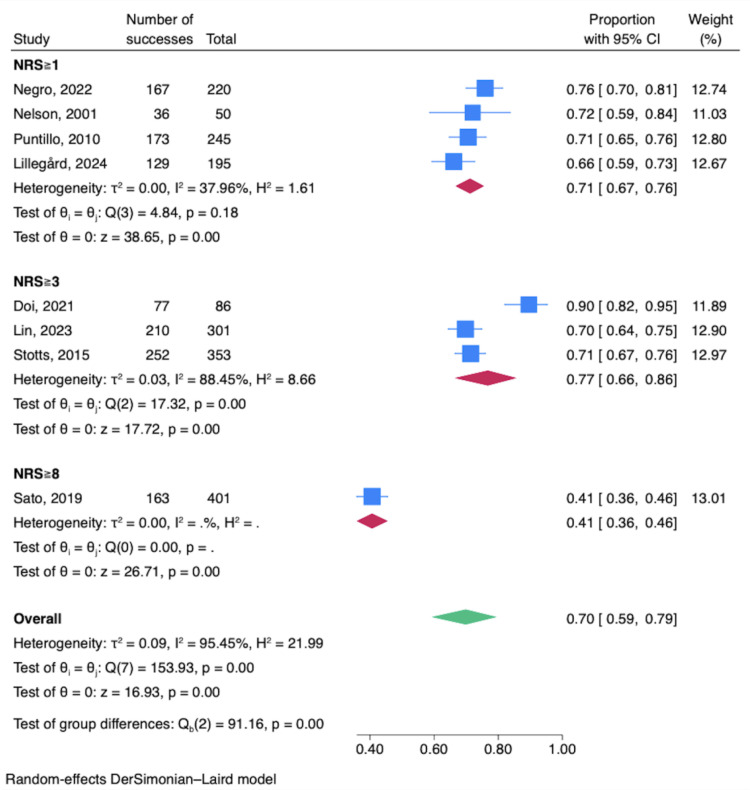
Forest plot for prevalence of thirst sensation.

The prevalence of thirst is summarized in Fig 2. Excluded studies in this analysis are summarized in S1 and S2 Tables. Eight studies [4,6,12,15,17–20] reported the prevalence of thirst drawing on data from 2,204 patients, resulting in a prevalence estimate of 0.70 (95% CI: 0.59–0.79), with high statistical heterogeneity (T^2^ =  0.09, I^2^ =  95.45%, P < .001). When stratified by the definition of thirst, there were four references with a numerical rating scale (NRS) score ≥  1 or with other scales to determine the presence of thirst. The prevalence estimate was 0.71 (95% CI: 0.67–0.76), with low statistical heterogeneity (T^2^ =  0.00, I^2^ =  37.96%, P = .18). Three references with an NRS score ≥  3 had a prevalence estimate of 0.77 (95% CI: 0.66–0.86), with high statistical heterogeneity (T^2^ =  0.03, I^2^ =  88.45%, P < .001). One had an NRS score ≥  8 and a prevalence estimate of 0.41 (95% CI: 0.36–0.46); heterogeneity could not be assessed. For excluded study is summarized in S4 File.

The risk factors for thirst are summarized in Table 3. Risk factors for thirst were divided into patient and treatment factors. Four patient factors were identified: serum sodium concentration, hyperglycemia, illness severity, and xerostomia. Six treatment factors were identified: nil per os, surgery, diuretics, humidified Venturi mask, serotonin reuptake inhibitors, and high doses of opioids in morphine equivalents (>50 mg).

**Table 3:** Risk factors for thirst in critically ill patients.

Patient factors	Treatment factors
Serum sodium concentration	Nil per os
Hyperglycemia	Surgery
Illness severity	Diuretic
Xerostomia	Humidified venturi mask
	Serotonin reuptake inhibitors
	High doses of opioids in morphine equivalents ( > 50 mg)

### Secondary outcomes

The methods used to evaluate thirst are summarized in Table 4. Thirteen studies reported methods of assessing thirst, with NRS being the most frequently evaluated method by nine references [6,7,8,13,15,17,18,23]. The visual analog scale (VAS) was reported by one study [14], the Edmonton Symptom Assessment Scale by two studies [4,12], the Vidatak EZ picture board by one study [16], Patient Symptom Survey symptom checklist [19] and the Tobii Dynavox I-15 eye tracking device by one study [16].

**Table 4:** Assessment method and definition of thirst sensation included in the systematic review.

Measurement tool	Scale	Thirst definition	Number of studies
NRS	0–10 point scale	None, NRS ≥ 1, NRS ≥ 3, NRS ≥ 8	9
Visual analog scale	0–100 or 0–10 point scale	None	1
Edmonton Symptom Assessment Scale	Yes/no response	Yes	2
Patient Symptom Survey symptom checklist	Yes/no response	Yes	1
Vidatak EZ picture board	Yes/no response	Yes	1
The Tobii Dynavox I-15 eye tracking device	Follow a cursor with their eyes		1

*Note:* NRS, numerical rating scale

Symptom relief methods for thirst are summarized in Table 5. Five articles reported methods for alleviating the symptoms of thirst. Doi et al. [15] examined changes in the NRS for thirst, lip moistness, and the Modified Revised Oral Assessment Guide after providing oral care every four hours to the intervention group. The results showed a significant effect on the NRS of thirst immediately after oral care (-1 [-3 to 0], P < .01) to 1 h later (0 [-1.25 to 1], P =  0.04). Jang et al. [21] performed tooth brushing, swabbing with 0.1% chlorhexidine, and intermittent swabbing with cold water for the intervention group and conventional oral care using gauze and saline for the target group, and compared them with the Beck Oral Health Assessment Scale. The results showed significant effects on oral status (8.1 vs. 11.1, P < .001), dry mouth (sublingual: 2.0, 1.0, P < .001; dorsal tongue: 1.0, 0.0, P < .001), and pH (6.5, 5.7, P < .001). Puntillo et al. [7] compared the intensity of thirst and distress in an intervention group with 15 min of oral swabbing, water spray, and application of menthol moisturizer to patients’ lips and in a control group with 15 min of usual care. The results showed significant effects on both thirst intensity (-2.3 vs. -0.6) and distress (-1.8 vs. -0.4). In a RCT [8], the intervention group received hourly oral moisturization with vitamin C spray, mouthwash with peppermint water, and application of lip balm. Oral moisturizer with saline spray every hour, mouthwash with warm water, and lip moisturizer with warm water every 2 h were given to the control group, and NRS of thirst feeling and the objective oral mucosa scale were compared. The study results showed significant effects on both the NRS of thirst (-1.27 vs. -0.19, P < .001) and the objective oral mucosa scale (-0.36 vs. 0.1, P = .008). In addition, there were four references that used oral care bundles and interventions. On the other hand, Merliot-Gailhoustet et al. [22], who assessed pain, anxiety, thirst, dyspnea, and insomnia with virtual reality equipment and music therapy for the intervention group and usual care, such as television and radio for the target group, did not find a significant reduction in thirst.

**Table 5:** Symptom relief methods of thirst in critically ill patients.

Author_ year	Sample size	Intervention group	Control group	Intervention	Comparator
Doi S_2021	86			Oral care every 4 hours	None
Jang CS_2016	35	18	17	Twice a day at 05:00 and 17:00. Oral care Swabbing with 0.1% chlorhexidine Intermittent swabbing with cold water	The control group received conventional oral care with gauze and saline twice daily (at 05:00 and 17:00)
Merliot-Gailhoustet L_2022	60	HEALTHY-MIND© VR system: 53 DEEPSEN© VR system:56 MUSIC-CARE: 55	Normal Care: 54	Virtual reality devices and music therapy	TV and radio
Puntillo KA_2014	252	127	125	Apply menthol moisturizer to patients’ lips using an oral swab and water spray for 15 minutes	15 minutes of normal care
Zhang W_2022	61	31	30	Intervention was performed from 8:00 to 18:00 for three days Moisturized oral cavity with vitamin C spray (concentration: 10 mg/mL) every hour, mouth rinse with peppermint water (5 g of peppermint + 50 mL of slightly warm water cooled to 40°C) at 14:00, and apply moisturizer (main ingredient: glycerin) to lips	Intervention was performed from 8:00 to18:00 for three days. Saline spray every hour to moisturize the oral cavity, mouthwash (50 mL of hot water cooled to 40°C), moisturizing the mouth with slightly warm water every 2 hours, and routine oral care consisted of wiping with a saline cotton ball and rinsing with warm water

### Meta-regression analysis

Meta-regression analyses indicated no significant difference in the prevalence of thirst based on mean age, sex ratio, and mechanical ventilator use (S3 Table). We found a negative association between the overall prevalence of thirst and ICU length of stay.

### Publication bias

The funnel plot is shown in S1 Fig. However, Egger’s test results did not uncover any significant indications of publication bias (z =  1.38, p = .17).

## Discussion

A systematic review and meta-analysis of critically ill patients admitted to the ICU synthesized the prevalence of thirst in critically ill patients quantitatively from 14 references and qualitatively synthesized it regarding assessment, risk factors, and symptom relief methods. There were seven references on prevalence, indicating a very high prevalence rate of 70%. The most common evaluation method was the NRS, and the cut-off values for thirst differed in different studies. Furthermore, some studies classified and evaluated thirst sensation based on distress and intensity. There were seven references on risk factors, categorized into patient and treatment factors. Among the items listed as risk factors, serum sodium level, severity of illness, nil per os, diuretic administration, and tracheal intubation were the most common and highly relevant for critically ill patients. There were five references on symptom relief methods, all showing significant results related to oral care. Some references examined interventions to promote oral moistening and salivation using oral care bundles.

### Prevalence of thirst in critically ill patients

The prevalence of thirst in critically ill patients was very high in this study. To our knowledge, this is the first systematic review to focus on the prevalence of thirst in critically ill patients. Konstam et al. [23] reported a 16% prevalence of thirst in medically treated patients with stable heart failure. Lee et al. [24] also reported a prevalence of moderate to severe thirst of 55.8% in postoperative patients. Compared to these studies, the prevalence of thirst in critically ill patients was higher.

Mechanisms can be considered regarding risk factors for thirst sensation in critically ill patients. The reasons for thirst in critically ill patients include an environment in which they cannot drink freely, such as during fasting, as well as a deficiency of intracellular fluid due to elevated plasma osmolality, extracellular fluid due to decreased blood volume, and mouth dryness [25–28]. An increase in osmolality is believed to cause the release of antidiuretic hormones from the posterior pituitary gland by stimulation of osmotic receptors in the anteroventral wall of the third ventricle of the hypothalamus, which sends stimuli to the anterior cingulate cortex and cerebellum, causing a feeling of thirst. ICU patients are prone to hyperglycemia [29], hypernatremia has been reported in approximately 26% of ICU patients [30], and diuretics are associated with increased osmolality. As for the decreased blood volume, stimulation of the arterial and baroreceptors of the heart and kidneys, resulting in atrial natriuretic peptide inhibition and sodium and water reabsorption, may send stimuli to the anterior cingulate cortex and cerebellum, causing a sense of thirst. Generally, ICU patients are prone to thirst sensations due to fluid balance changes, such as bleeding, vomiting, diarrhea, sweating, and diuresis. When the mouth is opened by oral tracheal intubation, xerostomia may cause the secretion of antidiuretic hormone from peripheral osmotic receptors in the oropharynx, which sends stimuli to the anterior cingulate cortex and cerebellum, causing a sensation of thirst. Regarding opioid effects, which are associated with the body’s water regulation system and may affect thirst perception [31,32], selective serotonin reuptake inhibitor suppress electrical activity in the subfornical organ at the entrance to the third ventricle; therefore, these drugs may act on this forebrain structure to promote sodium intake and thirst [33].

Regarding humidified Venturi masks, there is the impression that heating and humidifying is a positive effect, however, these masks are a risk factor for thirst sensation. Although fasting, eating, drinking, and other factors may have an influence in the background, heating and humidifying alone is unlikely to lead to a reduction in thirst. Based on the above findings, it is likely that the difference in prevalence is due to more factors that cause thirst in severely ill patients. Therefore, it is necessary to determine the prevalence of thirst at an early stage using appropriate assessment methods.

### The assessment methods of thirst in critically ill patients

Based on the study results, the cut-off for the presence or absence of thirst using the NRS varies from NRS 1 or higher, NRS 3 or higher, to NRS 8 or higher, and the definition of thirst is not well defined. Owing to the subjective nature of the symptoms, it is difficult to define a clear cut-off. In any case, the optimal assessment of symptoms should include intensity, frequency, and distress [34], and should quantify more than just the presence or absence of thirst. Furthermore, in a systematic review examining strategies in the management of postoperative thirst [35,36], the NRS or VAS was the most common method of assessment. Similar to pain, the NRS or VAS method for evaluating thirst, a subjective symptom, is considered the gold standard. Recent studies have attempted to quantify the distress caused by dry mouth in detail using the Thirst Discomfort Scale, a 12-item five-point scale [37]. Thus, it is important to appropriately assess thirst for early detection, prevention, and symptom relief in critically ill patients.

### The symptom relief methods of thirst in critically ill patients

The mechanism of thirst is thought to stem from a deficiency of extracellular and intracellular components and from stimuli sent from the oropharyngeal region to peripheral osmotic receptors. Studies examining the mechanism of thirst have reported [27] that cold stimulation of the oropharyngeal region is more useful than correction of dehydration in improving thirst. The results indicated that methods to alleviate thirst included oral moisturizing and ice water, citric acid, and ascorbic acid for pH adjustment, use of peppermint and menthol to provide a cool sensation, and lip moisturizing. Cold water ingestion may be more effective than room temperature water in relieving thirst as a stimulus to the oropharyngeal region [38,39], and ice water is representative of direct cold stimulation of the oropharynx. Other interventions, like citric acid and ascorbic acid, can promote salivation and alleviate thirst [40]. It has been confirmed that peppermint and menthol act directly on the oral mucosa and provide long-lasting cold stimulation, which promotes saliva secretion, retaining moisture in the mucous membrane, and alleviating the feeling of thirst [28,40]. Although the intervention methods are different, this intervention can achieve the effects of both cold stimulation and saliva secretion. Regarding lip moisturization, frequent glycerin application can maintain mucosal homeostasis [41]. Therefore, these mechanisms suggest that the symptom relief methods qualitatively integrated into this study were effective for thirst.

Furthermore, symptomatic relief from thirst requires a bundle of multiple interventions rather than a single one. In their study of symptom relief, Puntillo et al. [7] compared the intensity of thirst and distress in an intervention group with 15 min of oral swabbing, water spray, and application of menthol moisturizer to patients’ lips and in a control group with 15 min of usual care. They reported a 2.3 point reduction in thirst intensity and a 1.8 point reduction in thirst distress. In Zhang et al.’s study [8], the intervention group received hourly oral moisturization with vitamin C spray, mouthwash with peppermint water, and application of lip balm. Oral moisturizer with saline spray every hour, mouthwash with warm water, and lip moisturizer with warm water every 2 h were given to the control group. They reported a 1.27 point decrease in the NRS score for dry mouth. For patients experiencing pain, a decrease of approximately two points in the NRS score is considered clinically important [42]. Therefore, Puntillo et al. may highlight an effective intervention because it reduced the intensity of thirst by more than two points. However, a comparison of the post-care NRS scores between the intervention and control groups revealed a decrease of only 1.1 points. Thus, differences in intervention bundles, intervention procedures, timing of evaluation, inclusion and exclusion criteria, and other factors make it difficult to directly compare results across studies; however, no single bundled intervention would be able to accomplish much if it were less effective.

### Limitations and implications

A strength of this study is that it is the first systematic review and meta-analysis to validate thirst in critically ill patients, allowing us to estimate its prevalence. However, the results showed high heterogeneity in the prevalence of thirst among critically ill patients. We estimated high heterogeneity based on the definition of thirst, with the group defined as NRS 3 or higher showing the greatest variability. In addition, meta-regression analyses results indicated that only length of ICU stay showed significant differences. However, further research is needed to fully explain the heterogeneity. Possible reasons for this include differences in patient populations, assessment methods, timing, inclusion and exclusion criteria, and other factors. Therefore, caution should be exercised when interpreting the integrated results regarding the prevalence of thirst in critically ill patients. As a challenge for future research, new surveys and studies on this subject are needed to obtain a more comprehensive overview as relevant studies are scarce. Second, since the study results showed high heterogeneity in the prevalence of thirst in critically ill patients, discussions about the definition of thirst, sample size, and inclusion criteria should be considered. Third, the criteria for the presence of thirst are defined differently by different studies, and it is unclear which values are clinically meaningful. Therefore, basic research is needed to unify thirst definitions in the future.

## Conclusion

From the study results, it was established that 70% of critically ill patients experienced thirst. To minimize the distress caused by thirst, it is necessary to understand the pathophysiology and risk factors, properly assess the early detection of symptoms, and implement symptom relief using oral care bundles. Moreover, further investigations are needed to obtain a more comprehensive overview of thirst among critically ill patients.

## Supporting information

S1 File PROSPERO protocol. (PDF)

S2 File Preferred reporting items for systematic reviews and meta-analyses statement checklist. (PDF)

S3 File Search formulas. (PDF)

S4 File Quality assessment of studies. (PDF)

S1 Table Description of inclusion/exclusion articles. (PDF)

S2 Table Data extraction of included articles in the systematic review and meta- analysis. (PDF)

S3 Table Results of the meta-regression analysis. (PDF)

S1 Fig Funnel plot. (PDF)

## References

[1] Kalfon P, Mimoz O, Auquier P, Loundou A, Gauzit R, Lepape A, et al. Development and validation of a questionnaire for quantitative assessment of perceived discomforts in critically ill patients. Intensive Care Med. 2010;36(10):1751–8. doi: 10.1007/s00134-010-1902-9 20502874

[2] Dessotte CAM, Rodrigues HF, Furuya RK, Rossi LA, Dantas RAS. Stressors perceived by patients in the immediate postoperative of cardiac surgery. Rev Bras Enferm. 2016;69(4):741–50. doi: 10.1590/0034-7167.2016690418i 27508481

[3] Chanques G, Nelson J, Puntillo K. Five patient symptoms that you should evaluate every day. Intensive Care Med. 2015;41(7):1347–50. doi: 10.1007/s00134-015-3729-x 25758669

[4] Puntillo KA, Arai S, Cohen NH, Gropper MA, Neuhaus J, Paul SM, et al. Symptoms experienced by intensive care unit patients at high risk of dying. Crit Care Med. 2010;38(11):2155–60. doi: 10.1097/CCM.0b013e3181f267ee 20711069 PMC3377582

[5] Kjeldsen CL, Hansen MS, Jensen K, Holm A, Haahr A, Dreyer P. Patients’ experience of thirst while being conscious and mechanically ventilated in the intensive care unit. Nurs Crit Care. 2018;23(2):75–81. doi: 10.1111/nicc.12277 28124464

[6] Sato K, Okajima M, Taniguchi T. Association of persistent intense thirst with delirium among critically ill patients: a cross-sectional study. J Pain Symptom Manage. 2019;57(6):1114–20. doi: 10.1016/j.jpainsymman.2019.02.022 30831240

[7] Puntillo K, Arai SR, Cooper BA, Stotts NA, Nelson JE. A randomized clinical trial of an intervention to relieve thirst and dry mouth in intensive care unit patients. Intensive Care Med. 2014;40(9):1295–302. doi: 10.1007/s00134-014-3339-z 24894026 PMC4149585

[8] Zhang W, Gu Q, Gu Y, Zhao Y, Zhu L. Symptom management to alleviate thirst and dry mouth in critically ill patients: a randomised controlled trial. Aust Crit Care. 2022;35(2):123–9. doi: 10.1016/j.aucc.2021.04.002 34119409

[9] Page MJ, McKenzie JE, Bossuyt PM, Boutron I, Hoffmann TC, Mulrow CD, et al. The PRISMA 2020 statement: an updated guideline for reporting systematic reviews. BMJ. 2021;372:n71. doi: 10.1136/bmj.n71 33782057 PMC8005924

[10] Pollock D, Peters MDJ, Khalil H, McInerney P, Alexander L, Tricco AC, et al. Recommendations for the extraction, analysis, and presentation of results in scoping reviews. JBI Evid Synth. 2023;21(3):520–32. doi: 10.11124/JBIES-22-00123 36081365

[11] Sterne JAC, Savović J, Page MJ, Elbers RG, Blencowe NS, Boutron I, et al. RoB 2: a revised tool for assessing risk of bias in randomised trials. BMJ. 2019;366:l4898. doi: 10.1136/bmj.l4898 31462531

[12] Nelson JE, Meier DE, Oei EJ, Nierman DM, Senzel RS, Manfredi PL, et al. Self-reported symptom experience of critically ill cancer patients receiving intensive care. Crit Care Med. 2001;29(2):277–82. doi: 10.1097/00003246-200102000-00010 11246306

[13] Li DTY, Puntillo KA. Evaluations of physiologic and behavioral responses to noxious procedures in sedated critically ill adult patients. UC San Francisco Electronic Theses and Dissertations; 2007. https://escholarship.org/uc/item/8f01d2cp

[14] Siami S, Polito A, Porcher R, Hissem T, Blanchard A, Boucly C, et al. Thirst perception and osmoregulation of vasopressin secretion are altered during recovery from septic shock. PLoS One. 2013;8(11):e80190. doi: 10.1371/journal.pone.0080190 24223220 PMC3819281

[15] Doi S, Nakanishi N, Kawahara Y, Nakayama S. Impact of oral care on thirst perception and dry mouth assessments in intensive care patients: an observational study. Intensive Crit Care Nurs. 2021;66:103073. doi: 10.1016/j.iccn.2021.103073 34059413

[16] Duffy EI, Garry J, Talbot L, Pasternak D, Flinn A, Minardi C, et al. A pilot study assessing the spiritual, emotional, physical/environmental, and physiological needs of mechanically ventilated surgical intensive care unit patients via eye tracking devices, head nodding, and communication boards. Trauma Surg Acute Care Open. 2018;3(1):e000180. doi: 10.1136/tsaco-2018-000180 30246152 PMC6144907

[17] Negro A, Villa G, Greco M, Ciriolo E, Luraschi EL, Scaramuzzi J, et al. Thirst in patients admitted to intensive care units: an observational study. Ir J Med Sci. 2022;191(5):2283–9. doi: 10.1007/s11845-021-02817-7 34668107 PMC8526093

[18] Lin R, Li H, Chen L, He J. Prevalence of and risk factors for thirst in the intensive care unit: an observational study. J Clin Nurs. 2023;32(3–4):465–76. doi: 10.1111/jocn.16257 35199411 PMC10078651

[19] Saltnes-Lillegård C, Rustøen T, Beitland S, Puntillo K, Hagen M, Lerdal A, et al. Self-reported symptoms experienced by intensive care unit patients: a prospective observational multicenter study. Intensive Care Med. 2023;49(11):1370–82. doi: 10.1007/s00134-023-07219-0 37812229 PMC10622338

[20] Stotts NA, Arai SR, Cooper BA, Nelson JE, Puntillo KA. Predictors of thirst in intensive care unit patients. J Pain Symptom Manage. 2015;49(3):530–8. doi: 10.1016/j.jpainsymman.2014.07.001 25116914 PMC4324384

[21] Jang CS, Shin YS. Effects of combination oral care on oral health, dry mouth and salivary pH of intubated patients: a randomized controlled trial. Int J Nurs Pract. 2016;22(5):503–11. doi: 10.1111/ijn.12460 27349717

[22] Merliot-Gailhoustet L, Raimbert C, Garnier O, Carr J, De Jong A, Molinari N, et al. Discomfort improvement for critically ill patients using electronic relaxation devices: results of the cross-over randomized controlled trial E-CHOISIR (Electronic-CHOIce of a System for Intensive care Relaxation). Crit Care. 2022;26(1):263. doi: 10.1186/s13054-022-04136-4 36057612 PMC9440448

[23] Konstam MA, Gheorghiade M, Burnett JC Jr, Grinfeld L, Maggioni AP, Swedberg K, et al. Effects of oral tolvaptan in patients hospitalized for worsening heart failure: the EVEREST Outcome Trial. JAMA. 2007;297(12):1319–31. doi: 10.1001/jama.297.12.1319 17384437

[24] Lee C-W, Liu S-T, Cheng Y-J, Chiu C-T, Hsu Y-F, Chao A. Prevalence, risk factors, and optimized management of moderate-to-severe thirst in the post-anesthesia care unit. Sci Rep. 2020;10(1):16183. doi: 10.1038/s41598-020-73235-5 32999369 PMC7527446

[25] Arai S, Stotts N, Puntillo K. Thirst in critically ill patients: from physiology to sensation. Am J Crit Care. 2013;22(4):328–35. doi: 10.4037/ajcc2013533 23817822 PMC3718499

[26] Thelin WR, Brennan MT, Lockhart PB, Singh ML, Fox PC, Papas AS, et al. The oral mucosa as a therapeutic target for xerostomia. Oral Dis. 2008;14(8):683–9. doi: 10.1111/j.1601-0825.2008.01486.x 19193197

[27] Figaro MK, Mack GW. Regulation of fluid intake in dehydrated humans: role of oropharyngeal stimulation. Am J Physiol. 1997;272(6 Pt 2):R1740-6. doi: 10.1152/ajpregu.1997.272.6.R1740 9227585

[28] Arai SR, Butzlaff A, Stotts NA, Puntillo KA. Quench the thirst: lessons from clinical thirst trials. Biol Res Nurs. 2014;16(4):456–66. doi: 10.1177/1099800413505900 24136996 PMC3989478

[29] Cely CM, Arora P, Quartin AA, Kett DH, Schein RMH. Relationship of baseline glucose homeostasis to hyperglycemia during medical critical illness. Chest. 2004;126(3):879–87. doi: 10.1378/chest.126.3.879 15364770

[30] Stelfox HT, Ahmed SB, Khandwala F, Zygun D, Shahpori R, Laupland K. The epidemiology of intensive care unit-acquired hyponatraemia and hypernatraemia in medical-surgical intensive care units. Crit Care. 2008;12(6):R162. doi: 10.1186/cc7162 19094227 PMC2646327

[31] Sanger DJ, McCarthy PS. Increased food and water intake produced in rats by opiate receptor agonists. Psychopharmacology (Berl). 1981;74(3):217–20. doi: 10.1007/BF00427097 6791229

[32] Spencer RL, Deupree D, Hsiao S, Mosberg HI, Hruby V, Burks TF, et al. Centrally-administered opioid selective agonists inhibit drinking in the rat. Pharmacol Biochem Behav. 1986;25(1):77–82. doi: 10.1016/0091-3057(86)90233-9 2875475

[33] de Magalhães-Nunes AP, Badauê-Passos D Jr, Ventura RR, Da Silva Guedes D Jr, Araújo JP, Granadeiro PC, et al. Sertraline, a selective serotonin reuptake inhibitor, affects thirst, salt appetite and plasma levels of oxytocin and vasopressin in rats. Exp Physiol. 2007;92(5):913–22. doi: 10.1113/expphysiol.2007.037358 17573416

[34] Dodd M, Janson S, Facione N, Faucett J, Froelicher ES, Humphreys J, et al. Advancing the science of symptom management. J Adv Nurs. 2001;33(5):668–76. doi: 10.1046/j.1365-2648.2001.01697.x 11298204

[35] Silva TTM da, Dantas JKDS, Araújo SCM de, Silva S de O, Dantas DV, Dantas RAN. Strategies for thirst management in postoperative adult patients: a scoping review. Rev Bras Enferm. 2022;75(4):e20220154. doi: 10.1590/0034-7167-2022-0154 36228294

[36] Wang R, Wang L, Liu T, Peng C. Effects of menthol on thirst during surgery patients fasting: a systematic review and meta-analysis of randomized controlled studies. Int J Nurs Pract. 2024;30(3):e13191. doi: 10.1111/ijn.13191 37582491

[37] Çiftçi B, Yıldız GN, Avşar G, Köse S, Aydın E, Doğan S, et al. Development of the thirst discomfort scale: a validity and reliability study. Am J Crit Care. 2023;32(3):176–83. doi: 10.4037/ajcc2023954 37121897

[38] Peyrot des Gachons C, Avrillier J, Gleason M, Algarra L, Zhang S, Mura E, et al. Oral cooling and carbonation increase the perception of drinking and thirst quenching in thirsty adults. PLoS One. 2016;11(9):e0162261. doi: 10.1371/journal.pone.0162261 27685093 PMC5042416

[39] van Belzen L, Postma EM, Boesveldt S. How to quench your thirst. The effect of water-based products varying in temperature and texture, flavour, and sugar content on thirst. Physiol Behav. 2017;180 45–52. doi: 10.1016/j.physbeh.2017.08.007 28811190

[40] Han P, Suarez-Durall P, Mulligan R. Dry mouth: a critical topic for older adult patients. J Prosthodont Res. 2015;59(1):6–19. doi: 10.1016/j.jpor.2014.11.001 25498205

[41] Kvalheim SF, Marthinussen MC, Haugen DF, Berg E, Strand GV, Lie S-A. Randomized controlled trial of the effectiveness of three different oral moisturizers in palliative care patients. Eur J Oral Sci. 2019;127(6):523–30. doi: 10.1111/eos.12655 31325345

[42] Farrar JT, Young JP Jr, LaMoreaux L, Werth JL, Poole MR. Clinical importance of changes in chronic pain intensity measured on an 11-point numerical pain rating scale. Pain. 2001;94(2):149–58. doi: 10.1016/S0304-3959(01)00349-9 11690728

