# Supplementary material for: Prevalence, risk factors, and treatment methods of thirst in critically ill patients: A systematic review and meta-analysis
Source: PLoS One. 2025 Mar 18;20(3):e0315500. doi: 10.1371/journal.pone.0315500 (PMC11918398; doi:10.1371/journal.pone.0315500)
Supplement: S1 File — (PDF) [file pone.0315500.s001.pdf]

# Prevalence, Risk Factor, and Treatment Options of Thirst in Critically Ill Patients: Systematic review and Meta-analysis

To enable PROSPERO to focus on COVID-19 submissions, this registration record has undergone basic automated checks for eligibility and is published exactly as submitted. PROSPERO has never provided peer review, and usual checking by the PROSPERO team does not endorse content. Therefore, automatically published records should be treated as any other PROSPERO registration. Further detail is provided here.

## Citation

Takuto Fukunaga, Akira Ouchi, Gen Aikawa, Hideaki Sakuramoto, Saiko Okamoto, Shogo Uno. Prevalence, Risk Factor, and Treatment Options of Thirst in Critically Ill Patients: Systematic review and Meta-analysis. PROSPERO 2023 CRD42023428619 Available from:
https://www.crd.york.ac.uk/prospero/display_record.php?ID=CRD42023428619

## Review question

What are the prevalence, risk factors, and treatment options of thirst in critically ill patients?

## Searches

We will search articles in three electronic database including MEDLINE via PubMed, Cochrane Library (CENTRAL), and CINAHL. In addition, we will hand search using Google Scholar. All the English publications until 1 June 2023 will be searched without any restriction of countries. Reference list of all selected articles will independently screened to identify additional studies left out in the initial search.

None

## Types of study to be included

1) Studies of cohort, cross-sectional study design, and prospective studies (Randomized Controlled Trial and Non Randomized Controlled Trial)

2) Studies reporting risk factors for thirst in critically ill patients

3) Studies reporting the prevalence of thirst in critically ill patients

4) Studies in the English language only

5) Studies of human subjects only (no pre-clinical results)

Exclusion: Unable to extract the required data; Cases, reports, conferences, reviews, etc.

## Condition or domain being studied

The prevalence of thirst in critically ill patients is high, with about 70% of ICU patients complaining of severe thirst in a survey on discomfort symptoms. Thirst causes great distress and stress and increases oxygen consumption and metabolic burden on organs. Thirst can also induce delirium, which can affect patient recovery. Therefore, it is important to assess thirst and intervene to alleviate symptoms. This study identified the prevalence, risk factor, and treatment of thirst through meta-analysis.

## Participants/population

Inclusion: Adult patients who are critically ill and expected to stay in the ICU for more than 24 hours

Exclusions: age < 18; Patients with disturbance of consciousness; unable to informed consent; desquamation on the mouth or lips; a medical condition that contraindicated the intervention

## Intervention(s), exposure(s)

Any type of treatment for thirst or dry mouth

## Comparator(s)/control

Usual care

## Context

The context of this review will be critically ill patients in intensive care unit.

## Main outcome(s)

Primary outcome: Prevalence of thirst and symptom relief methods

## Additional outcome(s)

Other outcomes will be included diagnosis criteria or screening tests.

## Measures of effect

None

## Data extraction (selection and coding)

Study selection: The screening of articles by title and abstract will be carried out independently by two researchers who will be blinded to the other's screening (TF, HS, SO, GA, and SU). Full-text papers will be then independently screened by the two researchers (TF, HS, SO, GA, and SU) using the eligibility criteria described above. Disagreements and discrepancies will be resolved through discussion and consensus with the other reviewers (AO).

Data extraction: Studies that focus on assessing the prevalence of thirst and factors associated with thirst were focused upon. Published studies obtained through the database searches will be exported and duplicate studies will be merged using the RAYYAN software. Further screened based on the eligibility criteria will be carried out to include only those considered relevant for the review and meta-analyses. Data will be extracted into Microsoft Excel. Each article will be summarized under the following data headings in a table on the Excel data spread sheet:• First author/year of publication• Title of Article• Demographic factors (including average age, sex),• Study characteristics (Study design, study year of publication)• control group(type)•Factors associated, nature of trauma, symptom profile(hospital setting/community• occupational factors •social support• Personality• Co-morbidities• other factors •Sample size• Outcome.

## Risk of bias (quality) assessment

The Cochrane Risk of Bias2 (RoB2: a revised tool for assessing risk of bias in randomised trials. Bmj. 2019;366:l4898) will be used to assess the quality of the study design and the degree of potential bias according to the domains of this bias tool. Two reviewers (TF, AO) will evaluate the risk of bias independently using the RoB2. Disagreements between the two reviewers will be discussed, and if this fails, a third reviewer (HS) will be acting as an arbiter, if necessary.

The quality of cohort studies was evaluated using Newcastle–Ottawa Scale (NOS). Two reviewers (TF, AO) will evaluate the risk of bias independently using the NOS.

## Strategy for data synthesis

Data will be abstracted into summary tables which will be used for qualitative (descriptive) analyses. We will undertake meta-analyses only where this was meaningful. Because it is anticipated that the studies will be highly heterogeneous, random-effects meta-analyses for prevalence will be conducted.

Evaluation of Heterogeneity:

We will evaluate the statistical heterogeneity by visual inspection of the forest plots and calculating the $I^2$ statistic to quantify the magnitude of heterogeneity between studies($I^2$ values of 0% to 40%: might not be important; 30%to 60%: may represent moderate heterogeneity; 50% to 90%: may represent substantial heterogeneity; 75% to 100%: considerable heterogeneity). If heterogeneity is detected ($I^2 > 50\%$), we examine its causes: a Cochrane $\chi^2$ test (Q-test) is performed for the $I^2$ value, with a P-value <0.10 considered statistically significant. If significant heterogeneity is found, the median of the estimates is reported, not the weighted estimates.

Assessment of reporting biases:

If more than 10 studies are included in this review, we will perform a funnel plot analysis and will use Egger's test (p-value of < 0.05 being considered to be statistically significant for a two-sided test) to assess reporting bias.

## Analysis of subgroups or subsets

We plan to carry out the following subgroup analyses for the primary outcomes.

□Participants subsets: mechanically ventilated patients, sepsis or not

## Contact details for further information

Takuto Fukunaga

takuto.fukunaga@ns.toho-u.ac.jp

## Organisational affiliation of the review

None

None

## Review team members and their organisational affiliations

Mr Takuto Fukunaga. Toho University Omori Medical Hospital

Dr Akira Ouchi. Ibaraki Christian University

Dr Gen Aikawa. Ibaraki Christian University

Dr Hideaki Sakuramoto. Japanese Red Cross Kyushu International College of Nursing

Ms Saiko Okamoto. Department of Emergency and Critical Care Medicine, Hitachi General Hospital

Mr Shogo Uno. Department of Emergency and Critical Care Medicine, Hitachi General Hospital

## Type and method of review

Meta-analysis, Systematic review

## Anticipated or actual start date

01 June 2023

## Anticipated completion date

31 March 2024

## Funding sources/sponsors

None

## Conflicts of interest

None known

## Language

English

## Country

Japan

## Stage of review

Review Ongoing

## Subject index terms status

Subject indexing assigned by CRD

## Subject index terms

Critical Care; Critical Illness; Humans; Prevalence; Risk Factors; Thirst

## Date of registration in PROSPERO

06 June 2023

## Date of first submission

26 May 2023

## Details of any existing review of the same topic by the same authors

No existing reviews.

## Stage of review at time of this submission

The review has not started

| Stage | Started | Completed |
|---|---|---|
| Preliminary searches | No | No |
| Piloting of the study selection process | No | No |
| Formal screening of search results against eligibility criteria | No | No |
| Data extraction | No | No |
| Risk of bias (quality) assessment | No | No |
| Data analysis | No | No |

*The record owner confirms that the information they have supplied for this submission is accurate and complete and they understand that deliberate provision of inaccurate information or omission of data may be construed as scientific misconduct.*

*The record owner confirms that they will update the status of the review when it is completed and will add publication details in due course.*

## Versions

06 June 2023

06 June 2023
