# Supplementary material for: Prevalence, risk factors, and treatment methods of thirst in critically ill patients: A systematic review and meta-analysis
Source: PLoS One. 2025 Mar 18;20(3):e0315500. doi: 10.1371/journal.pone.0315500 (PMC11918398; doi:10.1371/journal.pone.0315500)
Supplement: S3 File — (PDF) [file pone.0315500.s003.pdf]

S3 File: Search formulas.

PubMed

#1 "critical care"[MeSH Terms]
#2 "critical illness"[MeSH Terms]
#3 "intensive care units"[MeSH Terms]
#4 "suction"[MeSH Terms]
#5 "critical care"[Title/Abstract]
#6 "critical illness"[Title/Abstract]
#7 "intensive care unit*"[Title/Abstract]
#8 "mechanical ventilation"[Title/Abstract]
#9 "ventilated patient*"[Title/Abstract]
#10 "critical illness"[Title/Abstract] OR "intensive care unit*"[Title/Abstract] OR "mechanical ventilation"[Title/Abstract] OR "ventilated patient*"[Title/Abstract]
#11 "thirst"[MeSH Terms]
#12 "thirst*"[Title/Abstract]
#13 "dry mouth"[Title/Abstract]
#14 "cold oral stimuli"[Title/Abstract]
#15 "ice"[Title/Abstract]
#16 "cold water"[Title/Abstract]
#17 "gargling"[Title/Abstract]
#18 "hydrator"[Title/Abstract]
#19 "wet gauze"[Title/Abstract]
#20 "wipe*"[Title/Abståract]
#21 "menthol*"[Title/Abstract]
#22 "menthol*"[Title/Abstract]
#23 "lip moisturizer"[Title/Abstract]
#24 "Xerostomia"[Title/Abstract]
#25 "Xerostomia"[MeSH Terms]
#26 "thirst"[MeSH Terms] OR "thirst*"[Title/Abstract] OR "dry mouth"[Title/Abstract] OR "cold oral stimuli"[Title/Abstract] OR "ice"[Title/Abstract] OR "cold water"[Title/Abstract] OR "gargling"[Title/Abstract] OR "hydrator"[Title/Abstract] OR "wet gauze"[Title/Abstract] OR "swab*"[Title/Abstract] OR "wipe*"[Title/Abstract] OR "citric acid spray*"[Title/Abstract] OR

"menthol*"[Title/Abstract] OR "lip moisturizer"[Title/Abstract] OR "Xerostomia"[Title/Abstract] OR "Xerostomia"[MeSH Terms]

#27 ("thirst"[MeSH Terms] OR "thirst*"[Title/Abstract] OR "dry mouth"[Title/Abstract] OR "cold oral stimuli"[Title/Abstract] OR "ice"[Title/Abstract] OR "cold water"[Title/Abstract] OR "gargling"[Title/Abstract] OR "hydrator"[Title/Abstract] OR "wet gauze"[Title/Abstract] OR "swab*"[Title/Abstract] OR "wipe*"[Title/Abstract] OR "citric acid spray*"[Title/Abstract] OR "menthol*"[Title/Abstract] OR "lip moisturizer"[Title/Abstract] OR "Xerostomia"[Title/Abstract] OR "Xerostomia"[MeSH Terms]) AND ("critical care"[MeSH Terms] OR "critical illness"[MeSH Terms] OR "intensive care units"[MeSH Terms] OR "suction"[MeSH Terms] OR "critical care"[Title/Abstract] OR "critical illness"[Title/Abstract] OR "intensive care unit*"[Title/Abstract] OR "mechanical ventilation"[Title/Abstract] OR "ventilated patient*"[Title/Abstract])

Cochrane Library(CENTRAL)

#1 MeSH descriptor: [Critical Care] explode all trees
#2 MeSH descriptor: [Critical Illness] explode all trees
#3 MeSH descriptor: [Intensive Care Units] explode all trees
#4 ("mechanical ventilation"):ti,ab,kw
#5 ("critical care"):ti,ab,kw
#6 ("critical illness"):ti,ab,kw
#7 ("intensive care unit*"):ti,ab,kw
#8 ("ventilated patient"):ti,ab,kw
#9 #1 OR #2 OR #3 #4 OR #5 OR #6 OR #7 OR #8
#10 MeSH descriptor: [Thirst] explode all trees
#11 MeSH descriptor: [Xerostomia] explode all trees
#12 ("thirst"):ti,ab,kw
#13 ("dry mouth"):ti,ab,kw
#14 ("Xerostomia"):ti,ab,kw
#15 ("cold oral stimuli"):ti,ab,kw
#16 ("cold water"):ti,ab,kw
#17 ("ice"):ti,ab,kw
#18 ("gargling"):ti,ab,kw
#19 ("hydrator"):ti,ab,kw

#20 ("wet gauze"):ti,ab,kw
#21 ("swab"):ti,ab,kw
#22 ("wipe"):ti,ab,kw
#23 ("citric acid spray"):ti,ab,kw
#24 ("menthol"):ti,ab,kw
#25 ("lip moisturizer"):ti,ab,kw
#26 #10 OR #11 OR #12 OR #13 OR #15 OR #16 OR #17 OR #18 OR #19 OR #20 OR #21 OR #22 OR #23 OR #24 OR #25
#27 #9 AND #26

CINAHL

((MH thirst+) OR (MH xerostomia+) OR (TI thirst* OR AB thirst*) OR (TI "dry mouth" OR AB "dry mouth") OR (TI "cold oral stimuli" OR AB "cold oral stimuli") OR (TI ice OR AB ice) OR (TI "cold water" OR AB "cold water") OR (TI gargling OR AB gargling) OR (TI hydratorOR AB hydrator) OR (TI "wet gauze" OR AB "wet gauze") OR (TI wipe* OR AB wipe*) OR (TI "citric acid spray*" OR AB "citric acid spray*") OR (TI menthol* OR AB menthol*) OR (TI "lip moisturizer" OR AB "lip moisturizer")) AND ((MH "critical care+") OR (MH "critical illness+") OR (MH "intensive care units+") OR (MH suction+) OR (TI "critical care" OR AB "critical care") OR (TI "critical illness" OR AB "critical illness") OR (TI "intensive care unit*" OR AB "intensive care unit*") OR (TI "mechanical ventilation" OR AB "mechanical ventilation") OR (TI "ventilated patient*" OR AB "ventilated patient*"))
