# Supplementary material for: Prevalence, risk factors, and treatment methods of thirst in critically ill patients: A systematic review and meta-analysis
Source: PLoS One. 2025 Mar 18;20(3):e0315500. doi: 10.1371/journal.pone.0315500 (PMC11918398; doi:10.1371/journal.pone.0315500)

S4 File: Quality assessment of studies.

Newcastle-Ottawa Scale

Author	Selection				Comparability		Outcome		
	Representativeness of the exposed	Selection of the non exposed cohort	Ascertainment of exposure	Demonstration that outcome of interest was not present at start of study	Comparability of cohorts on the basis of the design or analysis Age	Comparability of cohorts on the basis of the design or analysis Other controlled	Assessment of outcome	Was follow-up long enough for outcomes to occur	Adequacy of follow up of cohorts
Nelson JE_2001	☆	-	-	-	☆	☆	☆	-	-
Li DTY_2007	☆	☆	-	-	☆	☆	☆	-	-
Puntillo KA_2010	☆	☆	-	☆	☆	☆	☆	-	-
Siami S_2013	☆	☆	-	-	☆	☆	☆	☆	☆
Doi S_2021	☆	☆	-	-	☆	☆	☆	☆	☆
Duffy EI_2018	☆	☆	-	-	☆	☆	☆	-	-
Stotts NA_2015	☆	☆	-	-	☆	☆	☆	-	-
Sato K_2019	☆	☆	-	-	☆	☆	☆	-	-
Negro A_2022	☆	☆	-	-	-	-	☆	-	-
Lin R_2023	☆	☆	-	-	☆	☆	☆	-	-
Saltnes-Lillegård C_2024	☆	☆	-	☆	☆	☆	☆	-	-

Cochrane Risk of Bias 2

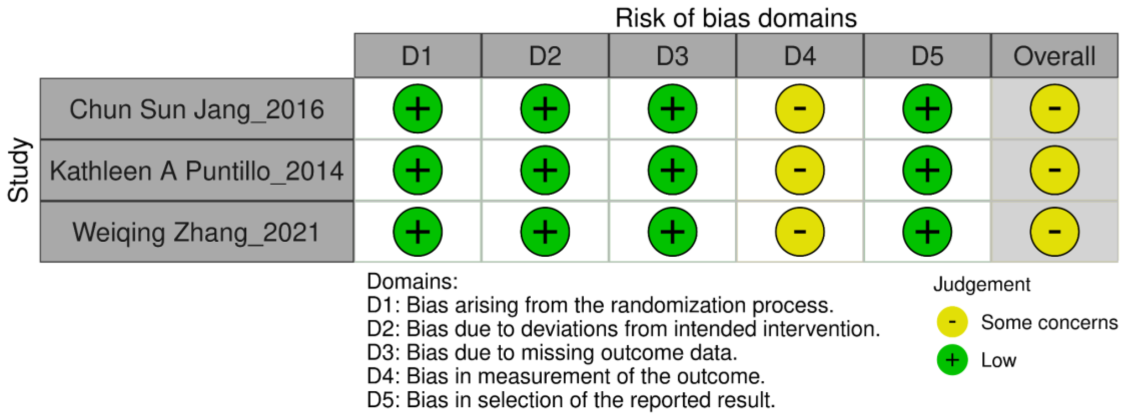

Cochrane Risk of Bias 2 tool for cross-over trials

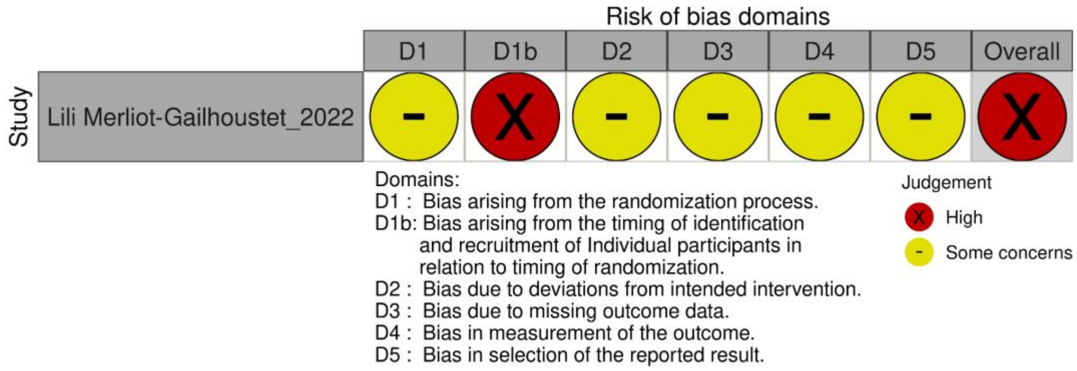

Supplement: S4 File — (PDF) [file pone.0315500.s004.pdf]
