# Supplementary material for: Prevalence, risk factors, and treatment methods of thirst in critically ill patients: A systematic review and meta-analysis
Source: PLoS One. 2025 Mar 18;20(3):e0315500. doi: 10.1371/journal.pone.0315500 (PMC11918398; doi:10.1371/journal.pone.0315500)
Supplement: S1 Table — (PDF) [file pone.0315500.s005.pdf]

S1 Table: Description of inclusion/exclusion articles.

no	Study reference	Title	Included /Excluded	Reasons for exclusion	Link to Unpublished Studies
1	Leemhuis A et al,2019	Palliation of Thirst in Intensive Care Unit Patients: Translating Research Into Practice	Excluded	Wrong study design	N/A
2	Aliti G B et al, 2013	Aggressive fluid and sodium restriction in acute decompensated heart failure: a randomized clinical trial	Excluded	Wrong outcome	N/A
3	Aliti G B et al, 2013	Aggressive fluid and sodium restriction in acute decompensated heart failure: a randomized clinical trial	Excluded	Duplicate records removed	N/A
4	Nascimento A D et al, 2013	SURGICAL PATIENT THIRST: ESTABLISHMENT AND VALIDATION OF A PROTOCOL FOR SAFELY MANAGING THIRST	Excluded	Wrong outcome	N/A
5	Anderson J et al,1999	Can a patient who has an endotracheal tube and is on mechanical ventilation be given ice chips?	Excluded	Wrong study design	N/A
6	Anggraeni D T, 2022	The effect of oral care using honey as an additional topical agent on oral health status of intubated patients in the intensive care unit	Excluded	Wrong outcome	N/A
7	Arai S et al, 2013	Thirst in critically ill patients: from physiology to sensation	Excluded	Wrong study design	N/A
8	Arai S et al, 2013	Thirst in Critically Ill Patients: From Physiology to Sensation	Excluded	Duplicate records removed	N/A
9	Pereira A et al, 2012	Assessment strategies for the management of thirst in the post-anesthetic recovery room	Excluded	Wrong population	N/A
10	Arora S K et al, 2013	Hypernatremic disorders in the intensive care unit	Excluded	Wrong study design	N/A
11	Ayllón Garrido N et al, 2007	Stressful environmental events in the Intensive Care Unit	Excluded	Wrong study design	N/A

12	Pereira E B F et al, 2021	Conhecimento, práticas e métodos para o alívio da sede no pós-operatório imediato entre profissionais de enfermagem	Excluded	Foreign language	N/A
13	Baumstarck K et al, 2019	Assessment of patients' self-perceived intensive care unit discomforts: Validation of the 18-item version of the IPREA	Excluded	Wrong outcome	N/A
14	Baumstarck K et al, 2019	Assessment of patients' self-perceived intensive care unit discomforts: validation of the 18-item version of the IPREA	Excluded	Duplicate records removed	N/A
15	Baumstarck K et al, 2019	Assessment of patients' self-perceived intensive care unit discomforts: validation of the 18-item version of the IPREA	Excluded	Duplicate records removed	N/A
16	Baumstarck K et al, 2019	Assessment of patients' self-perceived intensive care unit discomforts: Validation of the 18-item version of the IPREA	Excluded	Duplicate records removed	N/A
17	Belete K G et al, 2022	Prevalence and factors associated with thirst among postsurgical patients at University of Gondar comprehensive specialized hospital. Institution-based cross-sectional study	Excluded	Wrong population	N/A
18	Rhonda B, 2019	Clinical Pearls	Excluded	Wrong publication type	N/A
19	Rhonda B, 2023	Clinical Pearls	Excluded	Wrong publication type	N/A
20	Cain C, 2019	Frequency of Oral Care to Address Thirst: A Practice in Search of Evidence	Excluded	Wrong study design	N/A
21	Cazorla C et al, 2007	[Patients' perception of their experience in the intensive care unit]	Excluded	Wrong study design	N/A
22	Gu Y, 2019	Study for nursing effect of secondary dry mouth disease in critical patients in ICU	Excluded	Wrong study design	N/A
23	Cochran J et al, 1989	A Comparison of nurses' and patients' perceptions of intensive care unit stressors	Excluded	Wrong outcome	N/A

24	Cochran J et al, 1989	A Comparison of nurses' and patients' perceptions of intensive care unit stressors	Excluded	Duplicate records removed	N/A
25	Curtis J R et al, 2014	The critical importance of symptoms during and after intensive care	Excluded	Wrong study design	N/A
26	Dale C M et al, 2020	Exploration of difficulty accessing the mouths of intubated and mechanically ventilated adults for oral care: A video and photographic elicitation study	Excluded	Wrong study design	N/A
27	Dennesen P et al, 2003	Inadequate salivary flow and poor oral mucosal status in intubated intensive care unit patients	Excluded	Wrong outcome	N/A
28	Dessotte C A et al, 2016	Stressors perceived by patients in the immediate postoperative of cardiac surgery	Excluded	Wrong outcome	N/A
29	Doi S et al, 2021	Impact of oral care on thirst perception and dry mouth assessments in intensive care patients: An observational study	Included		N/A
30	Duffy E I et al, 2018	A pilot study assessing the spiritual, emotional, physical/environmental, and physiological needs of mechanically ventilated surgical intensive care unit patients via eye tracking devices, head nodding, and communication boards	Included		N/A
31	Estaji Z et al, 2015	The Comparison of Chlorhexidine Solution and Swab With Toothbrush and Toothpaste Effect on Preventing Oral Lesions in Hospitalized Patients in Intensive Care Unit	Excluded	Wrong outcome	N/A
32	Flim M et al, 2022	Measuring thirst distress of patients in the intensive care unit	Excluded	Wrong study design	N/A
33	Ford C et al, 2020	Safety and Effectiveness of Early Oral Hydration in Patients After Cardiothoracic Surgery	Excluded	Wrong study design	N/A
34	Ford S J et al, 2008	The importance and provision of oral hygiene in surgical patients	Excluded	Wrong study design	N/A
35	Garcia A K A et al, 2023	Implementation of the thirst management model in the burn unit guided by knowledge translation	Excluded	Wrong study design	N/A

36	Gültekin Y et al, 2018	Evaluation of stressors in intensive care units	Excluded	Wrong outcome	N/A
37	Halm M A, 2022	Managing Thirst in the Critically Ill	Excluded	Wrong publication type	N/A
38	Hammond L et al, 2023	Relieving Perception of Thirst and Xerostomia in Patients with Palliative and End-of-life Care Needs: A Rapid Review	Excluded	Wrong study design	N/A
39	Haubertin C et al, 2016	[Measuring the sources of discomfort in patients in intensive care]	Excluded	Foreign language	N/A
40	Hensen J, 2012	[Hyponatremia : The water-intolerant patient]	Excluded	Foreign language	N/A
41	Shari H, 2019	Aloe Vera Inner Leaf Gel and Peppermint Essential Oil Preparation Improves Oral Health of Patients in Hospital Intensive Care Units	Excluded	Wrong study design	N/A
42	Hiesmayr M J et al, 2012	PP043-SUN HUNGER AND THIRST IN THE ICU: BURDEN OR SYMPTOM IN THE NUTRITIONDAY ICU SURVEY?	Excluded	Wrong publication type	N/A
43	Holm A et al, 2017	Intensive care unit patients' experience of being conscious during endotracheal intubation and mechanical ventilation	Excluded	Wrong study design	N/A
44	Rezayi K, 2012	Comparison of the effect of echinacea and chlorhexidine mouthwashes on oral health of intubated patients in the Intensive Care Unit	Excluded	Wrong study design	N/A
45	Hajiabadi F, 2022	The effect of implementing a thirst relief program on the thirst in intensive care unit patients	Excluded	Wrong study design	N/A
46	Jang C S et al, 2016	Effects of combination oral care on oral health, dry mouth and salivary pH of intubated patients: a randomized controlled trial	Included		N/A
47	Jo Y M et al, 2015	A Comparison of Different Application Times of Oral Care on Colonies of Microorganisms and Oral Health Status on Intubated Patients	Excluded	Foreign language	N/A

48	Kalfon P et al, 2010	Development and validation of a questionnaire for quantitative assessment of perceived discomforts in critically ill patients	Excluded	Wrong outcome	N/A
49	Kalfon P et al, 2010	Development and validation of a questionnaire for quantitative assessment of perceived discomforts in critically ill patients	Excluded	Duplicate records removed	N/A
50	Kalowes P G, 2007	Symptom burden at end of life in patients with terminal and life-threatening illness in intensive care units	Excluded	Wrong study design	N/A
51	Hacer K et al, 2021	Environmental stressors perceived by patients in the surgical intensive care unit and their level of satisfaction with nursing care	Excluded	Wrong outcome	N/A
52	Kumar N, 2020	Effectiveness of nocturnal dexmedetomidine nebulization for the prevention of delirium in ICU patient: a randomized, placebo-controlled trial	Excluded	Wrong interventio n	N/A
53	VonStein M et al, 2019	Effect of a Scheduled Nursing Intervention on Thirst and Dry Mouth in the Intensive Care Patients	Excluded	Wrong study design	N/A
54	Landströ m M et al, 2009	Perceptions of registered and enrolled nurses on thirst in mechanically ventilated adult patients in intensive care units-a phenomenographic study	Excluded	Wrong study design	N/A
55	Landströ m M et al, 2009	Perceptions of registered and enrolled nurses on thirst in mechanically ventilated adult patients in intensive care units--a phenomenographic study	Excluded	Duplicate records removed	N/A
56	Leemhuis A et al, 2019	Palliation of Thirst in Intensive Care Unit Patients: Translating Research Into Practice	Excluded	Wrong study design	N/A
57	Leemhuis A et al, 2019	Palliation of Thirst in Intensive Care Unit Patients: translating Research Into Practice	Excluded	Duplicate records removed	N/A
58	Li D T et al, 2006	A pilot study on coexisting symptoms in intensive care patients	Excluded	Wrong study design	N/A
59	Li D T et al, 2007	Evaluations of physiologic and behavioral responses to noxious procedures in sedated critically ill adult patients	Included		N/A

60	Liang T et al, 2022	Efficacy and Safety of Oral Hydration 1 Hour After Extubation of Patients Undergoing Cardiac Surgery: a Randomized Controlled Trial	Excluded	Wrong population	N/A
61	Lin R et al, 2022	Effects of a spray-based oropharyngeal moisturising programme for patients following endotracheal extubation after cardiac surgery: a randomised, controlled three-arm trial	Excluded	Wrong population	N/A
62	Lin R et al, 2023	Prevalence of and risk factors for thirst in the intensive care unit: An observational study	Included		N/A
63	Lombardo V et al, 2013	How caregivers view patient comfort and what they do to improve it: a French survey	Excluded	Wrong outcome	N/A
64	Marosti Dessotte C A et al, 2016	Stressors perceived by patients in the immediate postoperative of cardiac surgery	Excluded	Wrong study design	N/A
65	Meriläinen M ea al, 2013	Patients' interactions in an intensive care unit and their memories of intensive care: a mixed method study	Excluded	Wrong study design	N/A
66	Merliot-Gailhoustet L et al, 2022	Discomfort improvement for critically ill patients using electronic relaxation devices: results of the cross-over randomized controlled trial E-CHOISIR (Electronic-CHOIce of a System for Intensive care Relaxation)	Included		N/A
67	Puntillo K A et al, 2008	ICU Patient and Family Comfort Study	Excluded	Wrong study design	N/A
68	Kalfon P et al, 2015	Reducing Discomforts in Critically Ill Patients: the IPREA3 Study	Excluded	Wrong study design	N/A
69	Türk G, 2021	Oral Care Frequency in Mechanically Ventilated Patients	Excluded	Wrong study design	N/A
70	Negro A et al, 2022	Thirst in patients admitted to intensive care units: an observational study	Included		N/A
71	Nelson J E et al, 2001	Self-reported symptom experience of critically ill cancer patients receiving intensive care	Included		N/A

72	Nelson J E et al, 2001	Self-reported symptom experience of critically ill cancer patients receiving intensive care	Excluded	Duplicate records removed	N/A
73	Puntillo K A et al, 2014	A randomized clinical trial of an intervention to relieve thirst and dry mouth in intensive care unit patients	Included		N/A
74	Puntillo K A et al, 2010	Symptoms experienced by intensive care unit patients at high risk of dying	Included		N/A
75	Ross S M et al, 2020	Aloe vera-Peppermint Gel (Veramin): An Effective Treatment for Mouth Dryness Among ICU Patients	Excluded	Wrong publication type	N/A
76	Russell M T et al, 1987	Nursing care plan. Compensating for xerostomia in the critically ill patient	Excluded	Wrong study design	N/A
77	Sato K et al, 2019	Association of Persistent Intense Thirst With Delirium Among Critically Ill Patients: A Cross-sectional Study	Included		N/A
78	Sato K et al, 2023	Effect of high-flow nasal cannula therapy on thirst sensation and dry mouth after extubation: A single-centre prospective cohort study	Excluded	Wrong population	N/A
79	Julie S et al, 2018	What's in this Issue	Excluded	Wrong publication type	N/A
80	Kapil S et al, 2020	Effectiveness of Thirst Bundle on Thirst and Dry Mouth among Patients Admitted in Intensive Care Units	Excluded	Wrong outcome	N/A
81	Siami S et al, 2013	Thirst perception and osmoregulation of vasopressin secretion are altered during recovery from septic shock	Included		N/A
82	Similowski T et al, 2022	Impact of emotional stimulations on thirst in critically ill mechanically ventilated patient	Excluded	Wrong study design	N/A
83	Simini B, 1999	Patients' perceptions of intensive care	Excluded	Wrong publication type	N/A
84	Smith T A et al, 2019	Respiratory Failure, Noninvasive Ventilation, and Symptom Burden: An Observational Study	Excluded	Wrong population	N/A

85	Stotts N A et al, 2015	Predictors of thirst in intensive care unit patients	Included		N/A
86	Treloar D M et al, 1995	Use of a clinical assessment tool for orally intubated patients	Excluded	Wrong outcome	N/A
87	VonStein M et al, 2019	Effect of a scheduled nurse intervention on thirst and dry mouth in intensive care patients	Excluded	Wrong study design	N/A
88	Wang Y et al, 2015	Analysis of Complaints from Patients During Mechanical Ventilation After Cardiac Surgery: A Retrospective Study	Excluded	Wrong outcome	N/A
89	Woodtli A O, 1990	Thirst: a critical care nursing challenge	Excluded	Wrong publication type	N/A
90	Yesilbalkan O U et al, 2019	Noninvasive Mechanical Ventilation Related Some Complications: Patients Treating Intensive Care Unit	Excluded	Wrong population	N/A
91	Zhang W et al, 2022	Symptom management to alleviate thirst and dry mouth in critically ill patients: a randomised controlled trial	Included		N/A
92	Hosseini S et al, 2024	Adherence to the Enhanced Recovery After Surgery in Cardiac Surgery Patients: A Randomized Clinical Trial	Excluded	Wrong population	N/A
93	Joohyun C et al, 2023	Anticholinergic Burden and Xerostomia in Critical Care Settings	Excluded	Wrong population	N/A
94	Joohyun C et al, 2023	Anticholinergic Burden and Xerostomia in Critical Care Settings	Excluded	Duplicate records removed	N/A
95	Gunnels M S et al, 2024	Assessing Discomfort in American Adult Intensive Care Patients	Excluded	Wrong interventio n	N/A
96	Gungor S et al, 2024	Effects of cold spray on thirst, frequency of oral care, and pain of general surgery intensive care unit patients	Excluded	Wrong population	N/A
97	Hung-Shen W U et al, 2024	The Comparative Effectiveness of Using Cold Water Oral Spray and Cold Saline Oral Spray for Thirst Relief in Patients With Endotracheal Intubation in the Intensive Care Unit	Excluded	Foreign language	N/A

98	Lian R et al, 2024	The effect of ice-cold water spray following the model for symptom management on postoperative thirst in patients admitted to intensive care unit-a randomized controlled study	Excluded	Wrong population	N/A
99	Lian R et al, 2024	The effect of ice-cold water spray following the model for symptom management on postoperative thirst in patients admitted to intensive care unit-a randomized controlled study	Excluded	Duplicate records removed	N/A
100	Saltnes-Lillegård C et al, 2024	Self-reported symptoms experienced by intensive care unit patients: a prospective observational multicenter study	Included		N/A
