# Supplementary material for: Prevalence, risk factors, and treatment methods of thirst in critically ill patients: A systematic review and meta-analysis
Source: PLoS One. 2025 Mar 18;20(3):e0315500. doi: 10.1371/journal.pone.0315500 (PMC11918398; doi:10.1371/journal.pone.0315500)
Supplement: S2 Table — (PDF) [file pone.0315500.s006.pdf]

S2 Table: Data extraction of included articles in the systematic review and meta- analysis.

no	Study reference	Title	Name of data extractors	Date of data extraction	Reason for inclusion	Extracted data
1	Doi S et al, 2021	Impact of oral care on thirst perception and dry mouth assessments in intensive care patients: An observational study	TF	June 16, 2023	Met all inclusion criteria.	Country: Japan<br>Study design: Cohort study<br>Purpose: To investigate the impact of oral care on the assessment of thirst and dry mouth in ICU patients<br>Patients: Patients ≧ 18 years of age<br>Meta-analysis data: 0.90 [0.82, 0.95]<br>Meta-regression analysis: mean age 70 years, sex ratio (male) 63%, mechanical ventilated patient 21%, ICU stay 4 days<br>Descriptive data: Thirst treatment method, Thirst assessment method
2	Duffy E I et al, 2018	A pilot study assessing the spiritual, emotional, physical/environmental, and physiological needs of mechanically ventilated surgical intensive care unit patients via eye tracking devices, head nodding, and communication boards	TF	June 16, 2023	Met all inclusion criteria.	Country: USA<br>Study design: Pilot prospective study<br>Purpose: Validate devices that aid communication, such as communication boards and eye tracking devices<br>Patients: Fitted with a ventilator, Speaks English, No cognitive impairment, No traumatic brain injury with motor impairment, Intubated patients over 20 years of age<br>Descriptive data: Thirst assessment method
3	Jang C S et al, 2016	Effects of combination oral care on oral health, dry mouth and salivary pH of intubated patients: a randomized controlled trial	TF	June 22, 2023	Met all inclusion criteria.	Country: South Korea<br>Study design: Randomized clinical trial<br>Purpose: To evaluate the combined effect of oral healthcare on oral health status, dry mouth, and salivary pH in critically ill and intubated patients<br>Patients: Intubated patients over 20 years of age<br>Descriptive data: Thirst treatment method
4	Li D T et al, 2007	Evaluations of physiologic and behavioral responses to noxious procedures in sedated critically ill adult patients	TF	June 22, 2023	Met all inclusion criteria.	Country: USA<br>Study design: Prospective, descriptive study<br>Purpose: To determine cardiovascular and pupillary reactivity and behavioral responses during distressing and non-distressing procedures in sedated ICU patients<br>Patients: Patients aged between 21 and 80 years, Patients on ventilators for more than 12 hours, Understand English,Ramsay score of 2<br>Descriptive data: Thirst assessment method

5	Lin R et al, 2023	Prevalence of and risk factors for thirst in the intensive care unit: An observational study	TF	June 22, 2023	Met all inclusion criteria.	Country: China<br>Study design: Prospective descriptive design<br>Purpose: To analyze the incidence of and factors contributing to dry mouth in ICU patients with and without dry mouth by analyzing differences in physiological, psychological, disease- and environment-related parameters<br>Patients: Patients 18 years of age or older, In the ICU for more than 24 hours, RASS of -1 to +1, Able to communicate verbally and understand the questionnaire, Consent to participate in the study<br>Meta-analysis data: 0.70 [0.64, 0.75]<br>Meta-regression analysis: mean age NA, sex ratio (male) 68.3%, mechanical ventilated patient 26.3%, ICU stay 6.5 days<br>Descriptive data: Thirst risk factor, Thirst treatment method, Thirst assessment method
6	Merliot-Gailhoustet L et al, 2022	Discomfort improvement for critically ill patients using electronic relaxation devices: results of the cross-over randomized controlled trial E-CHOISIR (Electronic-CHOIce of a System for Intensive care Relaxation)	TF	June 22, 2023	Met all inclusion criteria.	Country: France<br>Study design: Cross-over randomized controlled trial<br>Purpose: To examine the best ways to improve ICU patient discomfort among various electronic relaxation devices<br>Patients: Patients ≧ 18 years of age, CAM-ICU negative with RASS greater than or equal to 0, SOFA score of 3 or higher<br>Descriptive data: Thirst treatment method, Thirst assessment method
7	Negro A et al, 2022	Thirst in patients admitted to intensive care units: an observational study	TF	June 22, 2023	Met all inclusion criteria.	Country: Italy<br>Study design: Prospective observational study<br>Purpose: To investigate the incidence of thirst sensation in ICU patients and assess the association between dry mouth sensation and endotracheal tube, tracheostomy, spontaneous breathing, and oxygen therapy with or without humidification<br>Patients: Patients 18 years of age or older with tracheal intubation or tracheostomy and spontaneous respiration, GCS greater than or equal to 9<br>Meta-analysis data: 0.76 [0.70, 0.81]<br>Meta-regression analysis: mean age 61 years, sex ratio (male) 72.4%, mechanical ventilated patient NA, ICU stay 5.9 days<br>Descriptive data: Thirst risk factor, Thirst assessment method

8	Nelson J E et al, 2001	Self-reported symptom experience of critically ill cancer patients receiving intensive care	TF	June 22, 2023	Met all inclusion criteria.	Country: USA<br>Study design: Prospective observational study<br>Purpose: To clarify the experience of symptoms of ICU patients at high risk of in-hospital mortality<br>Patients: Cancer patients admitted to MICU<br>Meta-analysis data: 0.72 [0.59, 0.84]<br>Meta-regression analysis: mean age 65 years, sex ratio (male) 65%, mechanical ventilated patient 74%, ICU stay 5 days<br>Descriptive data: Thirst assessment method
9	Puntillo K A et al, 2014	A randomized clinical trial of an intervention to relieve thirst and dry mouth in intensive care unit patients	TF	June 13, 2023	Met all inclusion criteria.	Country: USA<br>Study design: Single-blinded, randomized clinical trial<br>Purpose: To examine the effects of intervention bundles on thirst intensity, thirst distress, and dry mouth<br>Patients: Patients aged over 18 years, Residing in the ICU for more than 24 hours, Speaks English, Can give name, date and location, RASS of -1 to +1, Either thirst intensity or thirst distress score at screening is NRS 3 or higher<br>Descriptive data: Thirst treatment method, Thirst assessment method
10	Puntillo K A et al, 2010	Symptoms experienced by intensive care unit patients at high risk of dying	TF	June 13, 2023	Met all inclusion criteria.	Country: USA<br>Study design: Prospective, observational study<br>Purpose: To assess the symptom experience of ICU patients at high risk of death and evaluate the relationship between delirium and patient symptoms<br>Patients: Patients 18 years or older admitted to the ICU for at least 3 days, APACHE II score of ≧ 20 in the first 24 hours, One or more of the following diagnoses: acute heart failure, respiratory failure, chronic liver failure with cirrhosis, multiorgan failure, sepsis, or systemic failure associated with a diagnosis of malignancy<br>Meta-analysis data: 0.71 [0.65, 0.76]<br>Meta-regression analysis: mean age 58 years, sex ratio (male) 64%, mechanical ventilated patient 72.4%, ICU stay NA<br>Descriptive data: Thirst assessment method
11	Sato K et al, 2019	Association of Persistent Intense Thirst With Delirium Among Critically Ill Patients: A Cross-sectional Study	TF	June 13, 2023	Met all inclusion criteria.	Country: Japan<br>Study design: Single-center retrospective cross-sectional study<br>Purpose: To determine if persistent severe thirst is strongly associated with the onset of delirium<br>Patients: Patients ≧ 18 years of age, RASS of -1 to +1<br>Meta-analysis data: 0.41 [0.36, 0.46]

						Meta-regression analysis: mean age 68.3 years, sex ratio (male) 59.6%, mechanical ventilated patient 57.3%, ICU stay NA<br>Descriptive data: Thirst assessment method
12	Siami S et al, 2013	Thirst perception and osmoregulation of vasopressin secretion are altered during recovery from septic shock	TF	June 13, 2023	Met all inclusion criteria.	Country: France<br>Study design: Prospective interventional study<br>Purpose: To examine how vasopressin secretion during an osmotic challenge (500 mL of hypertonic saline administered for 120 minutes) is a variable response in patients recovering from septic shock<br>Patients: Adult patients hospitalized with septic shock who survived five days after discontinuation of boosting agent therapy<br>Descriptive data: Thirst assessment method
13	Stotts N A et al, 2015	Predictors of thirst in intensive care unit patients	TF	June 13, 2023	Met all inclusion criteria.	Country: USA<br>Study design: Descriptive cross-sectional study<br>Purpose: To identify predictors of presence, intensity, and distress of dry mouth in ICU patients<br>Patients: Patients over 18 years of age, Residing in the ICU for more than 24 hours, Speaks English, Can give name, date, and location, RASS of -1 to +1<br>NRS of thirst is more than 3<br>Meta-analysis data: 0.71 [0.67, 0.76]<br>Meta-regression analysis: mean age 54.9 years, sex ratio (male) 54.7%, mechanical ventilated patient 4.8%, ICU stay 9.5 days<br>Descriptive data: Thirst risk factor
14	Zhang W et al, 2022	Symptom management to alleviate thirst and dry mouth in critically ill patients: a randomised controlled trial	TF	June 13, 2023	Met all inclusion criteria.	Country: China<br>Study design: Prospective, randomized, placebo-controlled<br>Purpose: To demonstrate the effectiveness of an intervention bundle to reduce thirst and dry mouth<br>Patients: 18 years of age or older, Treated in the ICU for more than 24 hours, Fasting patients, Clear conscious and able to cooperate, NRS of thirst measured by screening is greater than or equal to 3<br>Descriptive data: Thirst risk factor, Thirst treatment method, Thirst assessment method
15	Saltnes-Lillegård C et al, 2024	Self-reported symptoms experienced by intensive care unit patients: a prospective observational multicenter study	TF	June 12, 2024	Met all inclusion criteria.	Country: Norway<br>Study design: Prospective cohort study<br>Purpose: To describe the prevalence, intensity and distress of five symptoms in ICU patients and to investigate possible predictive factors associated with symptom intensity

						Patients: Patients 18 years of age or older, In the ICU for more than 24 hours, Need for mechanical ventilation, Need for continuous vasoactive therapy or ICU stay greater than 24 hours<br>Meta-analysis data: 0.66 [0.59, 0.73]<br>Meta-regression analysis: mean age 63.5 years, sex ratio (male) 59.3%, mechanical ventilated patient 31.7%, ICU stay 9.8 days<br>Descriptive data: Thirst assessment method
