# Supplementary material for: Prevalence, risk factors, and treatment methods of thirst in critically ill patients: A systematic review and meta-analysis
Source: PLoS One. 2025 Mar 18;20(3):e0315500. doi: 10.1371/journal.pone.0315500 (PMC11918398; doi:10.1371/journal.pone.0315500)
Supplement: S3 Table — (PDF) [file pone.0315500.s007.pdf]

S3 Table: Results of the meta-regression analysis.

	Number of studys	Coefficient	95% confidence interval	z value	p value
mean age	7	-0.003	-0.05, 0.05	-0.12	0.90
sex ratio	8	0.02	-0.03, 0.06	0.71	0.48
mechanical ventilator	7	-0.004	-0.01, 0.01	-0.85	0.40
ICU stay	6	-0.06	-0.11, -0.01	-2.23	0.03
