# Supplementary figures and images for: Prevalence, risk factors, and treatment methods of thirst in critically ill patients: A systematic review and meta-analysis
Source: PLoS One. 2025 Mar 18;20(3):e0315500. doi: 10.1371/journal.pone.0315500 (PMC11918398; doi:10.1371/journal.pone.0315500)

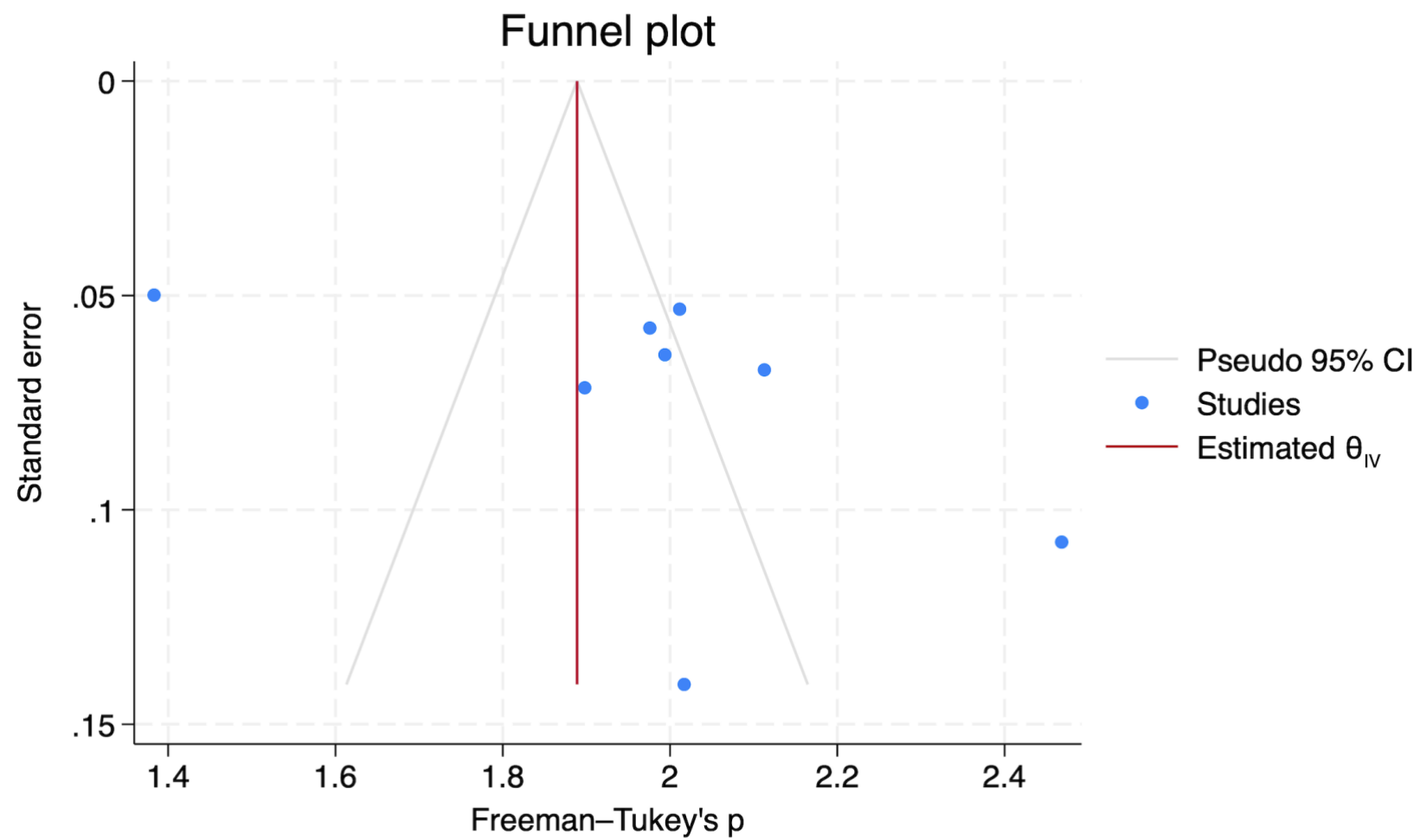

S1 Fig: Funnel plot.

Supplement: S1 Fig — (PDF) [file pone.0315500.s008.pdf]
