# Supplementary material for: Prevalence, risk factors, and treatment methods of thirst in critically ill patients: A systematic review and meta-analysis
Source: PLoS One. 2025 Mar 18;20(3):e0315500. doi: 10.1371/journal.pone.0315500 (PMC11918398; doi:10.1371/journal.pone.0315500)
Supplement: Attachment — Submitted filename: Manuscript Review.docx [file pone.0315500.s009.docx]

**Manuscript Review**

**Manuscript ID:** PONE-D-24-28619

**Title:** Prevalence, risk factors, and treatment methods of thirst in critically ill patients: A systematic review and meta-analysis

**Journal**: PLOS ONE

**Summary and general comments:**

In the manuscript “**Prevalence, risk factors, and treatment methods of thirst in critically ill patients: A systematic review and meta-analysis**” the authors performed a literature search followed by meta-analyses of 15 published studies.

The main results showed:

1. An overall pooled frequency of thirst estimated at 70%
2. A high overall between-studies heterogeneity (I^2^ = 95%) due, at least in part, to the definition of thirst

Overall, the paper is well written, straight forward, simple, clear and concise, and I really enjoyed reading it, however it needs some rectifications.

**Specific comments:**

**#1 Statistical analysis:** The statistical analysis section is incomplete and requires:

a) Definition of the effect size (proportion or logit transformed proportion, etc.) together with the Z test and the threshold of significance (presumably 0.05).

b) Analyzing between-studies heterogeneity through I^2^ is insufficient. I^2^ tell us nothing about the dispersion of effects across included studies. The authors should mention the Q test and the value of Tau^2^, and calculate the 95% prediction intervals (95% PI). **The authors should not confuse the 95% PI with the 95% CI.** The authors can use the CMA prediction intervals free software to compute all 95% PI through the following link <https://meta-analysis-workshops.com/pages/predictionintervals>. The calculation of the 95% PI is simple and based on the following 4 items: effect-size, upper bound of the 95% CI, Tau^2^ and number of included studies.

c) Publication bias assessment through Egger’s test and the generation of a funnel plot.

**#2** **Sources of heterogeneity**: The results showed that there was a huge amount of between-studies heterogeneity. The authors performed a subgroup analysis by “definition of thirst” based on a “numerical rating scale” which could explain at least in part the between studies heterogeneity. If possible/applicable the authors could improve their study through:

a- Subgroup analysis by ethnicity.
b- Meta-regressions using patients’ mean age, gender-ratio (M/F), condition, duration of hospital stay, and any relevant clinical or biological feature in critically ill patients.

This will enable the authors to enrich the results with more insightful information about sources of the observed heterogeneity.

**#3** **Publication bias**: The authors did not perform a publication bias analysis. The authors should:

a- Add a sub-chapter to the “statistical analysis” section, namely “Publication bias assessment” and describe the procedure. For instance: Publication bias was assessed by Egger’s test and visualized through the generation of a funnel plot.
b- Add a sub-chapter to the “Results” section in which the authors should depict the results of publication bias for each comparison.
c- Add a figure of a Funnel plot.

Thanks for the opportunity to review this manuscript. If appropriately corrected, this meta-analysis could provide valuable information on the frequency of thirst in critically ill patients, the risk factors and patients management.
